# Diverse Functions of IAA-Leucine Resistant PpILR1 Provide a Genic Basis for Auxin-Ethylene Crosstalk During Peach Fruit Ripening

**DOI:** 10.3389/fpls.2021.655758

**Published:** 2021-05-12

**Authors:** Xiaobei Wang, Junren Meng, Li Deng, Yan Wang, Hui Liu, Jia-Long Yao, Nicolaas Jacobus Nieuwenhuizen, Zhiqiang Wang, Wenfang Zeng

**Affiliations:** ^1^Zhengzhou Fruit Research Institute, Chinese Academy of Agricultural Sciences, Zhengzhou, China; ^2^College of Horticulture, Henan Agricultural University, Zhengzhou, China; ^3^The New Zealand Institute for Plant & Food Research Limited, Auckland, New Zealand

**Keywords:** *Prunus persica* (L. Batsch), auxin, ILR, ethylene, fruit ripening

## Abstract

Auxin and ethylene play critical roles in the ripening of peach (*Prunus persica*) fruit; however, the interaction between these two phytohormones is complex and not fully understood. Here, we isolated a peach *ILR* gene, *PpILR1*, which encodes an indole-3-acetic acid (IAA)-amino hydrolase. Functional analyses revealed that PpILR1 acts as a transcriptional activator of 1-amino cyclopropane-1-carboxylic acid synthase (*PpACS1*), and hydrolyzes auxin substrates to release free auxin. When Cys137 was changed to Ser137, PpILR1 failed to show hydrolase activity but continued to function as a transcriptional activator of *PpACS1* in tobacco and peach transient expression assays. Furthermore, transgenic tomato plants overexpressing *PpILR1* exhibited ethylene- and strigolactone-related phenotypes, including premature pedicel abscission, leaf and petiole epinasty, and advanced fruit ripening, which are consistent with increased expression of genes involved in ethylene biosynthesis and fruit ripening, as well as suppression of branching and growth of internodes (related to strigolactone biosynthesis). Collectively, these results provide novel insights into the role of IAA-amino acid hydrolases in plants, and position the PpILR1 protein at the junction of auxin and ethylene pathways during peach fruit ripening. These results could have substantial implications on peach fruit cultivation and storage in the future.

## Introduction

Ethylene plays a critical role in controlling the ripening climacteric fruits (Tucker et al., 2017). The ethylene biosynthesis pathway, originally described by Yang and colleagues (Yang and Hoffman, 1984; Grierson, 2013), involves two key steps: conversion of S-adenosyl methionine to 1-amino cyclopropane-1-carboxylic acid (ACC) by 1-amino cyclopropane-1-carboxylic acid synthase (ACS), and conversion of ACC to ethylene by ACC oxidase. ACS is unstable and exhibits very low levels in tissues that do not produce large amounts of ethylene; however, under conditions that promote ethylene biosynthesis, the activity of ACS is highly elevated. Therefore, ACS is generally considered as a commitment to ethylene biosynthesis and a rate limiting enzyme (Wang et al., 2002; Xu and Zhang, 2015).

Auxin has long been considered to counteract fruit ripening; however, a recent transcriptomic approach highlighted a previously underestimated role of auxin during regulation of fruit ripening in peach (*Prunus persica*) (Trainotti et al., 2007). Low levels of IAA lead to suppression of *PpACS1* expression at the late-ripening stage of stony hard peach, whereas high concentrations of IAA are required for ethylene biosynthesis, which results in rapid fruit softening (Tatsuki et al., 2013; Pan et al., 2015). However, since the auxin-ethylene relationship is very intricate, some of the phenotypic effects are yet to be assigned to either hormone.

The concentration of IAA in a plant is critical; low IAA concentrations positively impact plant physiological processes, but high concentrations are inhibitory and toxic. Plants coordinate IAA homeostasis by regulating its biosynthesis, transport, oxidation, degradation, and conjugation (Woodward and Bartel, 2005; Normanly, 2010; Korasick et al., 2013). Conjugation of auxin with other molecules facilitates storage of active IAA. Auxin conjugates can be low molecular weight (when conjugated with sugars or amino acid moieties via ester or amide bonds, respectively) or high molecular weight (when conjugated with peptides and proteins via amide bonds). Formation of IAA-amido conjugates inactivates IAA and also creates a reservoir of IAA that can be rapidly made available by IAA-amidohydrolases (Staswick et al., 2005).

In *Arabidopsis thaliana*, seven genes encoding IAA-amino acid hydrolase enzymes have been identified (LeClere et al., 2002). These enzymes display high affinity for IAA-Ala and IAA-Leu, but low affinity for other IAA-amino acid conjugates (Rampey et al., 2004; Korasick et al., 2013). Studies show that IAA-amido hydrolases regulate hypocotyl elongation, lateral root development, fungal infection response, and nodule formation in Arabidopsis, wheat (*Triticum aestivum*), *Brassica rapa*, and alfalfa (*Medicago truncatula*) (Rampey et al., 2004; Schuller and Ludwig-Müller, 2006; Campanella et al., 2008; Strader et al., 2010). In tomato (*Solanum lycopersicum*), changes in expression of the auxin-amido hydrolase gene *IAR3* perturb auxin homeostasis, which affects plant defense responses (Dippolito et al., 2016). However, the role of auxin-amido hydrolase enzymes in fruit ripening remains largely unknown.

In fruit crops such as apple (*Malus domestica*), tomato, and banana (*Musa* spp.), 1-methylcycolpropene (1-MCP), a strong ethylene binding inhibitor, is used widely to prolong the shelf-life of fruits and delay their ripening (Mathooko et al., 2001; Watkins, 2006). However, in peach, there have been contradictory reports; some researchers showed that 1-MCP blocks ethylene biosynthesis, thereby delaying fruit ripening (Mathooko et al., 2001; Bregoli et al., 2015), although not efficiently (Hayama et al., 2008), whereas others showed that 1-MCP enhances ethylene production (Dong et al., 2001; Dal et al., 2006; Ziliotto et al., 2008). Recently, Tadiello (Tadiello et al., 2016) showed that 1-MCP responses in melting flesh fruits vary according to the ripening stage. In peach fruit, 1-MCP is ineffective in delaying ripening because it stimulates an increase in free auxin levels, which lead to a burst in ethylene production, thereby speeding up the ripening process. This increase in the level of free auxin during the last stages of fruit ripening in peach is probably caused not only by its *de novo* biosynthesis but also by its release from conjugated forms (Tatsuki et al., 2013). Consistently, expression of the IAA-amido hydrolase gene (*CTG475*), which is involved in release of IAA from IAA-amino acid conjugates, correlates with free IAA content during ripening and following 1-MCP treatment (Tadiello et al., 2016).

Here, we identified the peach *PpILR1* gene, which primarily functions as a hydrolytic enzyme but also acts as a regulator of ethylene biosynthesis. Overexpression of *PpILR1* in tomato indicated multiple processes and pathways governing growth and reproduction. The results unravel the mechanistic crosstalk between auxin, ethylene, and strigolactone, revealing another dimension to the complex and dynamic phytohormone interaction.

## Materials and Methods

### Fruit Tissue Collection

Representatives of two peach cultivars with contrasting ripening behavior and flesh texture, “zhongyoutao13” (“CN13”; melting flesh (MF) type) and “zhongyoutao16” (“CN16”; stony hard (SH) type) (Zeng et al., 2015), grown in the research orchard of the Zhengzhou Fruit Research Institute, Zhengzhou, China, were used in this study. “CN13” produces MF-type fruits in which auxin and ethylene peak during ripening, whereas “CN16” produces SH-type fruits, which contain only basal levels of auxin and ethylene. “CN13” (MF) fruits were collected at 75, 80, 85, and 90 days after flowering (DAF) (designated as S3, S4 I, S4 II, and S4 III, respectively). “CN16” (SH) samples were collected at 70, 75, 80, and 85 DAF (designated as S3, S4 I, S4 II, and S4 III, respectively) as reported previously (Zeng et al., 2015). The fruit growth stage was defined according to Tonutti et al. (1997). Twenty fruits per sample were collected from five different trees. “CN13” fruits were harvested at the S4 II stage, which corresponds to class 1 (onset of climacteric) (Tadiello et al., 2016), and were treated with 1-MCP (10 μL/L) at 20°C for 1 d. “CN16” fruits were harvested at the S4 III stage (85 DAF) and treated with ethylene and NAA, as described by Zhu et al. (2017). The treated fruits were then stored at 20°C for 1, 2, 3, 4, or 5 d. Mesocarp tissues of fruits from different trees were collected, placed directly in liquid nitrogen, and stored in a freezer at −80°C until needed for RNA isolation.

### Ethylene Production and IAA Quantification

Ethylene production was measured as described by Zeng et al. (2015). Briefly, intact fruits were stored in an airtight container at 24°C for 2 h. Then, 1 ml of headspace was sampled and analyzed using GC2010 gas chromatograph. The ethylene content of each sample was measured in triplicate. The IAA content was measured according to Tatsuki et al. (2013). Briefly, 50 mg of powdered sample was mixed with 80% methanol, which contained 1% acetic acid (v/v). The slurry was incubated at 4°C for 10 min, and samples were then extracted with CH_2_Cl_2_ and analyzed by gas chromatography-mass spectrometry (GC-MS).

### Phylogenetic Analysis

Neighbor-joining phylogenetic trees, based on protein sequences and amino acid sequence alignment of PpILR1 and ILRs, were constructed using MEGA5.0 and Clustalx1.83. The deduced amino acid sequences of ILR proteins used to perform the phylogenetic analyses are listed in Supplementary Table 1.

### RNA Extraction, cDNA Synthesis, and Gene Expression Analysis

RNA extraction and cDNA synthesis were performed as described by Wang et al. (2017). RNA was extracted from peach and tomato fruit tissues using the Total RNA Kit (Tiangen, Beijing, China). RNA degradation and contamination were monitored by electrophoresis on 1% agarose gels, while RNA quality and quantity were assessed using NanoDrop ND-1000 spectrophotometer and an Agilent 2100 Bioanalyzer. Then, cDNA synthesis was performed using the FastKing RT Kit (Tiangen, Beijing, China). The relative expression level of *PpILR1* in each sample was analyzed by quantitative real-time PCR (qRT-PCR) using *Actin* (*ppa007242m*) (Tatsuki et al., 2013) as the internal control gene. The experiment was carried out as reported previously (Zeng et al., 2015). Primers used to perform qRT-PCR are listed in Supplementary Table 5. Three independent biological replicates were performed for each sample.

### Subcellular Localization of PpILR1

The subcellular localization of PpILR1 was examined as described previously (Feng et al., 2016; Liu et al., 2017). The open reading frame (ORF) of *PpILR1* was cloned into the pM999 and pCAMBIA1300 vectors. The nucleus was labeled with 4′,6-diamidino-2-phenylindole (DAPI). The green fluorescent protein (GFP) signal was observed using a fluorescence microscope (Leica SP8, Wetzlar, Germany) at 488-nm.

### Yeast One-Hybrid Assay (Y1H)

The coding sequence (CDS) of *PpILR1* was cloned into the pGADT7 vector. Full-length *PpACS1* promoter (0 to −1489 bp upstream of ATG) and different fragments of the *PpACS1* promoter, hereafter referred to as *PpACS1-P1* (−882 to −1489 bp), *PpACS1-P2* (−432 to −957 bp), and *PpACS1-P3* (−1 to −501 bp), were amplified and cloned into the pAbAi vector. The Matchmaker™ Gold Yeast One-Hybrid Library Screening System (Clontech, San Francisco, CA, USA) was used to verify the interaction between the PpILR1 transcription factor and *PpACS1* promoter fragments. Primers used for plasmid construction are listed in Supplementary Table 2.

### Dual-Luciferase Reporter Assay (DLR)

The *PpACS1* promoter fragments were cloned into the pGreenII 0800-LUC vector (Hellens et al., 2005), and the *PpILR1* CDS was cloned into the pK2GW7 vector under the control of the cauliflower mosaic virus (CaMV) 35S promoter. A dual-luciferase assay kit (Promega, Madison, USA) was used to measure firefly luciferase (LUC) and *Renilla* luciferase (REN) activities. The SpectraMax® i3x Platform (Molecular Devices, Sunnyvale, USA) was used to estimate absorbance. The LUC-to-REN ratio was determined. At least six measurements were obtained for each assay. Primers used for plasmid construction are listed in Supplementary Table 2.

### Transient Expression in Peach

The pK2GW7-PpILR1 expression construct was introduced into *Agrobacterium tumefaciens* strain GV3101, and the empty vector (pK2GW7) was used as a control. Peach fruits were harvested at the S3 stage (second exponential growth phase) and used for the transient expression assay, as described by Liu et al. (2017). Briefly, peach fruits were soaked for 20 min in sodium hypochlorite solution and washed three times with sterile water. Then, four 1-cm thick cubes of flesh were excised from the opposite sides of each fruit and pre-cultured for 24 h on Murashige and Skoog (MS) medium. Two cubes were infiltrated with *Agrobacterium* cultures carrying the 35S::PpILR1 construct, and the remaining two cubes were infiltrated with cultures carrying the empty vector (negative control).

### Electrophoretic Mobility Shift Assay (EMSA)

The *PpILR1* CDS was cloned into pGEX-6P-1 vector containing the GST tag, and the recombinant protein was purified as described by Wang et al. (2019). The purified PpILR1-GST fusion protein was used to perform the EMSA with the EMSA Kit (Thermo Scientific, Waltham, USA), according to the manufacturer's instructions. Sequence containing the GTGACA box, derived from the *PpACS1-P1* promoter fragment (Supplementary Table 3), were labeled with biotin at the 5′ terminus and used as probes. The same unlabeled DNA fragment was used as a competitor, while the GTGACA box within the probe changed to AAAAAA was used as the mutated probe in the assay.

### Site-Directed Mutagenesis

Site-directed mutagenesis was carried out using the recombinant plasmid pGEX-6P-1. The Cys137 residue, which is located in the PpILR1 enzyme binding pocket and is crucial for enzyme activity (Smolko et al., 2016), was changed to Ser137 using Fast Mutagenesis Kit V2 (Vazyme, Nanjing, China) according to the manufacturer's instructions. Briefly, point mutation of the *PpILR1* gene was carried out using sequence-specific primers (forward: TGCACGCAAGCGGCCATGATAGCCATGTTGCA; reverse: CATGGCCGCTTGCGTGCATTTTACCGTCAATTT) synthesized by Shenggong (Shanghai, China). Homologous recombination was used to construct the plasmid that contained the mutation site. PYMOL software was used to construct the PpILR1 model.

### Hydrolytic Activity of PpILR1

The hydrolysis activity assays of PpILR1 were performed in reactions containing 50 mM Tris-HCl (pH 8.0), 1 mM MnCl_2_, 1 mM dithiothreitol, 50 mM IAA-amino acid, and 2, 10, or 20 ng/ml PpILR1-GST fusion protein. Reactions were incubated at room temperature for 16–24 h. To determine whether any IAA was released from the conjugate, the reaction products were analyzed by GC-MS (Liu et al., 2018). IAA-Ala, IAA-Leu, and IAA were used as standard samples.

### Plant Transformation and Phenotypic Analysis

Since it is not feasible to stably transform peach plants, “Micro-Tom” tomato plants were transformed with the pK2GW7-PpILR1 and pK2GW7-PpILR1-M constructs via *Agrobacterium*-mediated transformation, as described by Wang et al. (2005). At least 20 fruits were harvested from each transgenic line (PpILR1-22, PpILR1-30) at the breaker (Br) stage, and at 1, 2, 3, and 6 days after the Br stage (Br+1, Br+2, Br+3, and Br+6, respectively), with three independent replicates. Ethylene production and fruit firmness were measured as described by Liu et al. (2014) and Ecarnot et al. (2013), respectively. To determine the expression levels of ethylene biosynthesis genes, sections <0.5-mm thick were excised from the flower abscission zone. To examine strigolactone biosynthesis gene expression analysis, only nodes were sampled. The harvested tissue samples were used for RNA extraction and cDNA synthesis. Expression levels of ripening-related genes, including ethylene biosynthesis genes, ripening regulators, and cell wall-related genes, were measured in the wild-type (WT) and transgenic tomato fruits by qRT-PCR, as described previously (Zouine et al., 2014), using *Solyc11g005330* as the internal reference gene. All gene expression analyses were performed with three independent biological replicates. Primers used to perform qRT-PCR are listed in Supplementary Table 4.

## Results

### PpILR1 Binds to the *PpACS1* Promoter

**Figure 1:**
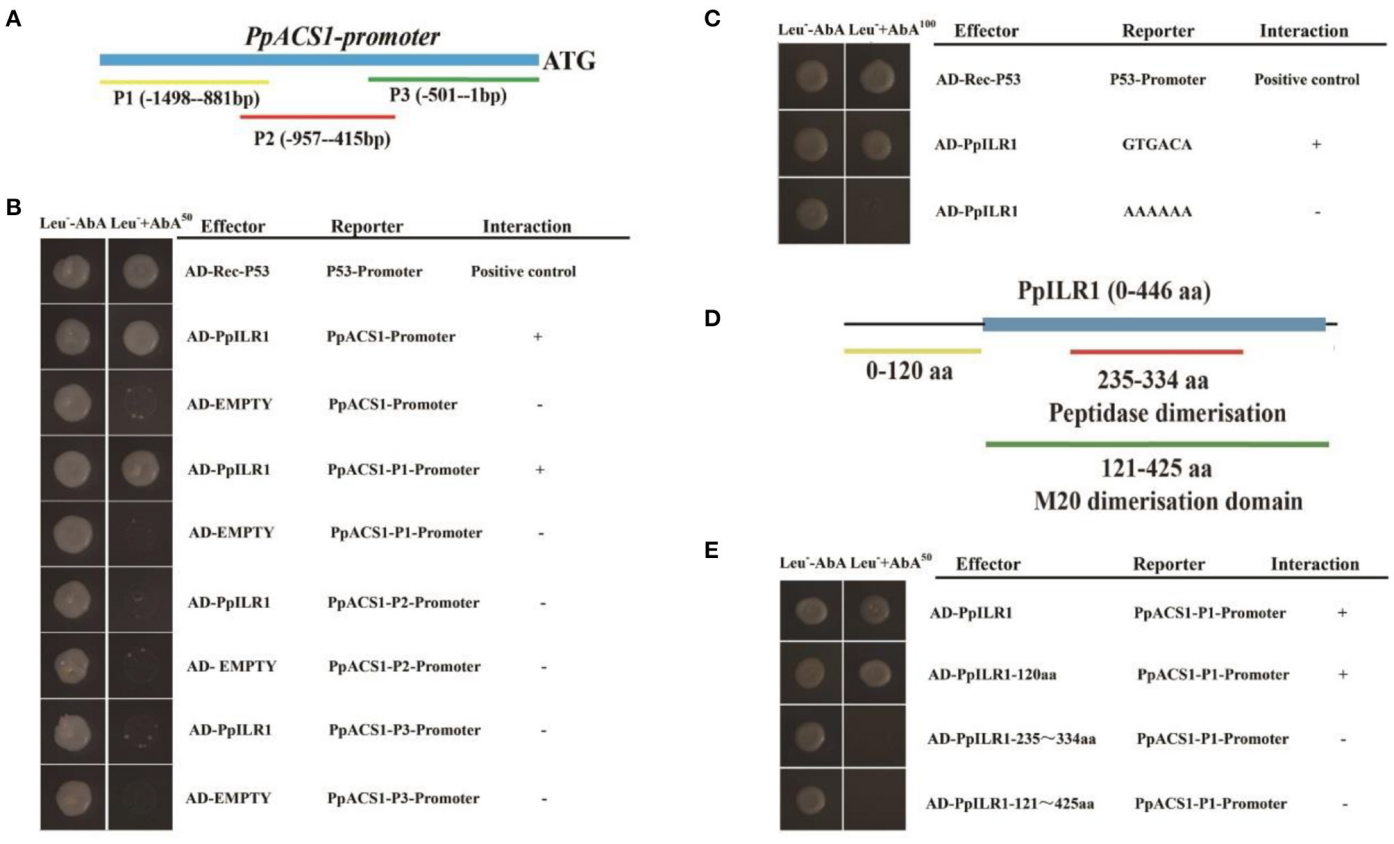
PpILR1 binds directly to the *PpACS1* promoter. **(A)** The *PpACS1* promoter was divided into three fragments. **(B)** Yeast one-hybrid (Y1H) analysis. Aureobasidin A (AbA; 50 ng/ml) was used as a selection marker. The Rec-P53 and P53 promoters were used as positive controls. The empty vector with the *PpACS1* promoter fragments was used as a negative control, and showed no interaction. **(C)** Y1H analysis showing that PpILR1 binds to the GTGACA element. **(D)** The PpILR1 protein was divided into three fragments. **(E)** Y1H assay showing that the PpILR1–120aa fragment (N-terminus) interacts with the *PpACS1-P1* promoter fragment.

Because PpACS1 is the rate limiting enzyme in the ethylene biosynthesis pathway, we used the pAbAi::PpACS1 construct to perform yeast one-hybrid library screening. A total of 32 proteins, including Prupe.7G100000 (PpILR1), were identified as candidates that potentially bind to *PpACS1* promoter sequences (Supplementary Table 5). To confirm the interaction between the *PpACS1* promoter and PpILR1 protein, the 1489-bp *PpACS1* promoter was divided into three fragments (*PpACS1-P1, PpACS1-P2*, and *PpACS1-P3*) (Figure 1A) and used to perform a new Y1H assay. The results showed that PpILR1 bound to the *PpACS1-P1* fragment (Figure 1B). An auxin response site, GTGACA (TGTCAC) (O'Malley et al., 2016), was found in the *PpACS1-P1* fragment. To investigate whether PpILR1 was able to bind to *PpACS1-P1* directly through the GTGACA motif, six repeats of the GTGACA motif were cloned into the Y1H reporter construct (Lopato et al., 2006). The results of Y1H assay showed that PpILR1 bound to tandem repeats of the GTGACA motif directly. When the GTGACA motif was mutated to poly-A sequence, no interaction was observed with the PpILR1 protein (Figure 1C). Furthermore, the GTGACA element was repeated six times and used as probe to confirm binding of PpILR1 to the *PpACS1* promoter through the GTGACA motif. A shift in the position of the DNA band was observed when PpILR1 was incubated with the GTGACA probe, but not when incubated with the mutant probe (where GTGACA was changed to AAAAAA) (Supplementary Figure 1). Moreover, to identify the PpILR1 domain that interacts directly with *PpACS1-P1*, the PpILR1 protein was divided into three fragments PpILR1-1–120aa (N-terminus), PpILR1-121–425aa (C-terminus), and PpILR1-235–334aa (middle region) (Figure 1D). In the Y1H assay, only PpILR1-1–120aa interacted with *PpACS1-P1* (Figure 1E), which suggested that PpILR1-1–120aa was the domain that directly interacted with *PpACS1-P1*.

### Characteristics of the *PpILR1* Gene and the Encoded Protein

Phylogenetic analysis of PpILR1 and ILL proteins from tomato and Arabidopsis showed that PpILR1 clustered with AtILR1 (Supplementary Figure 2). The amino acid sequence of PpILR1 was 65.37 and 60.97% similar to that of AtILR1 and SlILR1, respectively. Alignment of the deduced full-length amino acid sequences of ILRs and ILLs from peach and Arabidopsis revealed two conserved domains (M20 and M20 dimerization) (Supplementary Figure 3). The *PpILR1* gene amplified and cloned from peach showed an ORF of 1341 bp, which was predicted to encode a protein of 446 aa. The sequence of *PpILR1* amplified in this study was different from that in the peach reference genome by six SNPs. The last SNP (T to G at 1,306 bp) abolished the stop codon, thereby extending the protein from 435 to 446 aa (Supplementary Figure 4).

**Figure 2:**
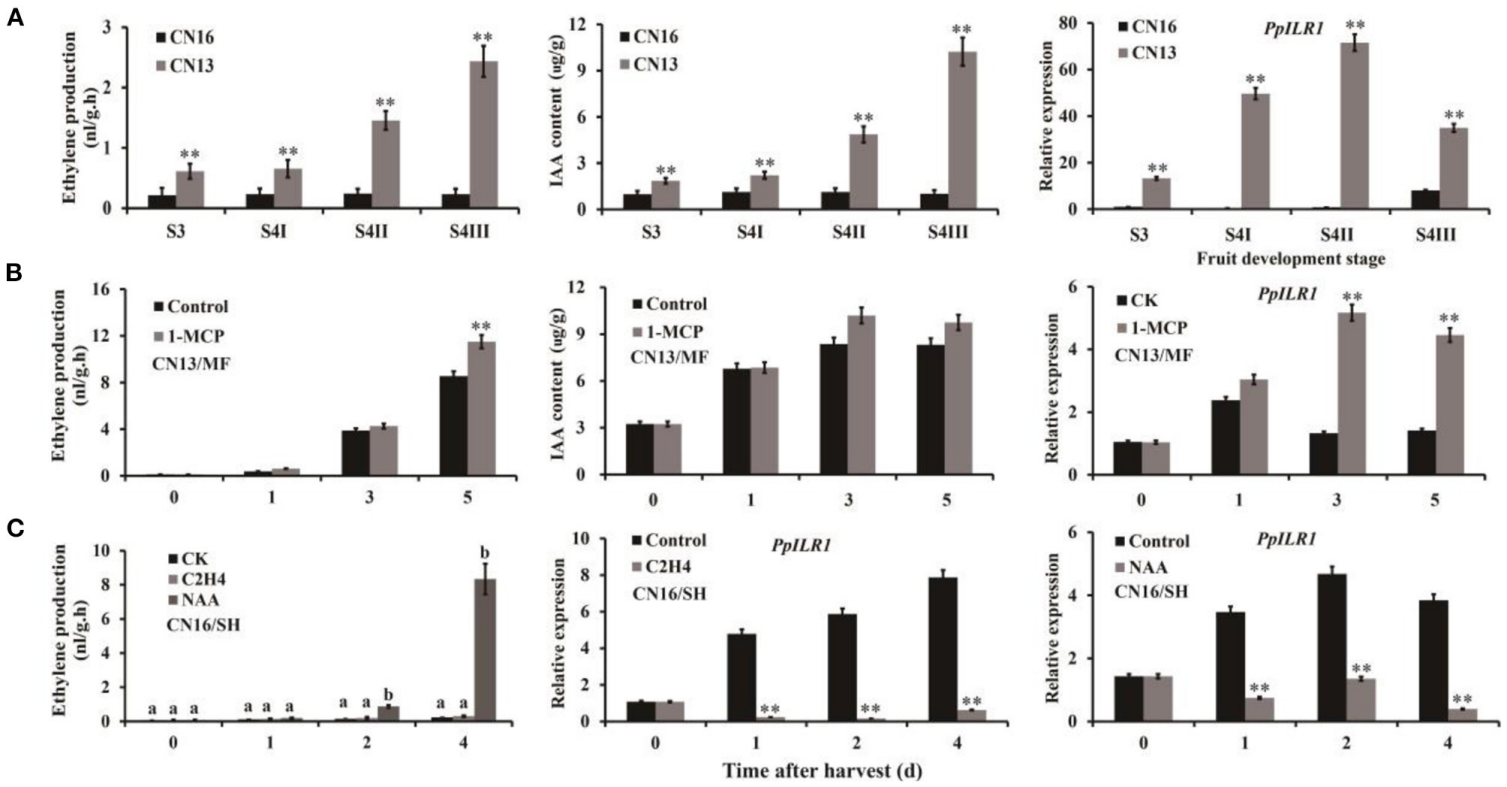
Expression pattern of *PpILR1*. **(A)** Ethylene and IAA content of “CN13” and “CN16” fruits during ripening, and the expression profile of *PpILR1* in “CN13” and “CN16” fruits. **(B)** Ethylene and IAA content of “CN13” fruits harvested at the S4 II stage after treatment with 1-MCP, and the expression level of *PpILR1*. **(C)** Endogenous ethylene production in “CN16” fruits harvested at the S4 III stage after treatment with NAA and ethylene, and the expression pattern of *PpILR1*. Values represent the mean of three biological replicates. Asterisks (*) indicate significant differences. Different letters indicate significant differences at *P* < 0.05.

The ethylene and IAA contents of “CN13” (MF) fruits were significantly higher than those of “CN16” (SH) fruits, and the expression level of *PpILR1* was significantly higher in “CN13” than in “CN16” fruits during ripening (Figure 2A), indicating that *PpILR1* is potentially involved in fruit ripening. Levels of ethylene and IAA, as well as the expression level of *PpILR1*, in “CN13” fruits increased after treatment with 1-MCP (Figure 2B). Compared with the control and ethylene-treated groups, ethylene production increased; but *PpILR1* expression was inhibited in “CN16” fruits treated with both ethylene and NAA (Figure 2C). To examine why 1-MCP treatment increased the IAA content of “CN13” fruits, we analyzed the expression pattern of auxin homeostasis-related genes in peach after treatment with 1-MCP (Supplementary Figure 5). The transcriptome data of these genes are summarized in Supplementary Table 6. Expression levels of *PpYUC11, PpACO1, PpGH3.1*, and *PpGH3.11* were not affected by 1-MCP treatment (Supplementary Figure 6), whereas those of *PpILR1* and *PpACS1* were increased significantly by 1-MCP (Figure 1B and Supplementary Figure 6). Because the PpILR1 enzyme increased the content of free auxin by hydrolyzing indole-3-acetic acid-amino acid (IAA-AA) conjugates (Smolko et al., 2016), the increase in *PpILR1* mRNA levels might contribute to the increase in the free IAA content. To determine the function of PpILR1, we analyzed the subcellular location of PpILR1. Transient expression of the *PpILR1-GFP* fusion in *Arabidopsis thaliana* protoplasts and tobacco leaves demonstrated that the PpILR1-GFP protein accumulates in the nucleus (Supplementary Figure 7).

### Cys137 Is Critical for the Hydrolytic Activity of PpILR1

The conserved Cys137 residue, located in the active site of the catalytic domain, plays an important role in regulation of enzyme activity (Smolko et al., 2016). To determine its importance for PpILR1 function, Cys137 was mutated to Ser137. The mutant protein, PpILR1-M, was predicted to have the same structure as the PpILR1 protein (Supplementary Figure 8A). In the PpILR1 and PpILR1-M proteins, the Mn^2+^ ion was predicted to be surrounded by Ser137, His139, Glu172, Glu173, and His197 residues (Supplementary Figures 8B,C), suggesting that substitution of Cys137 by Ser137 did not affect affinity of the protein for metal ions.

**Figure 3:**
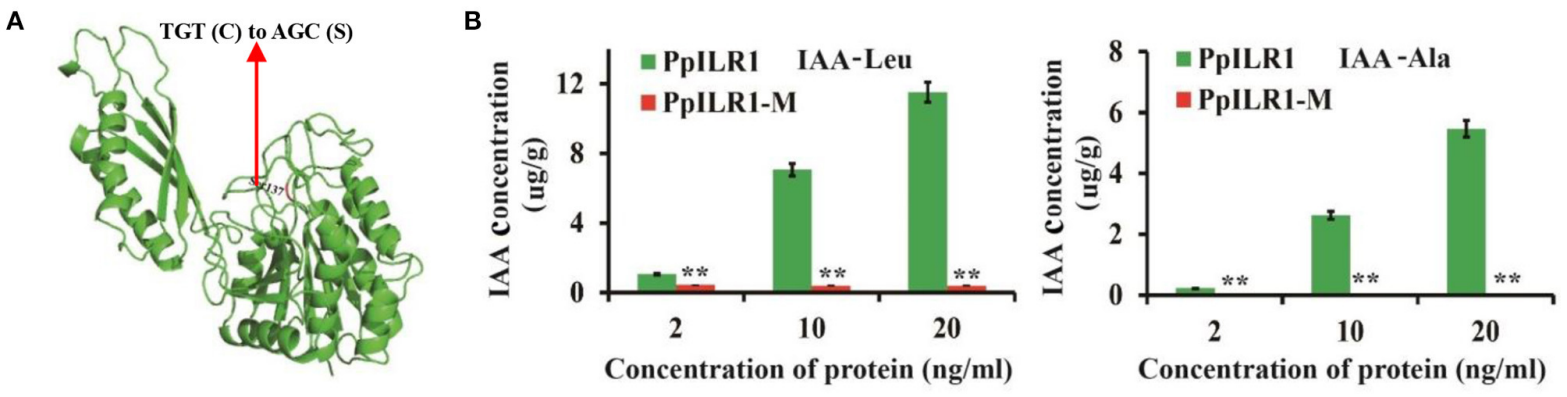
Analysis of the hydrolase activity of the wild-type (WT) protein PpILR1 and the mutant protein PpILR1-M. **(A)** Mutation site of PpILR1-M. The red arrow indicates the mutation of TGT (Cys137) to AGC (Ser137). **(B)** Analysis of the hydrolase activity of PpILR1 and PpILR1-M using IAA-Leu and IAA-Ala, respectively, as substrates. Free IAA was used as a standard. Asterisks indicate significant differences between the mutant and WT proteins (**P < 0.01; Student's *t*-test).

To determine whether the PpILR1-M protein retains the hydrolytic activity of the WT protein (Figure 3A), a hydrolysis experiment was carried out and the products were analyzed by GC-MS. The results showed that PpILR1 could hydrolyze both IAA-Ala and IAA-Leu and released free IAA. However, the mutant protein PpILR1-M exhibited almost no hydrolytic function (Figure 3B and Supplementary Table 7).

### PpILR1 and PpILR1-M Activate *PpACS1* Transcription

**Figure 4:**
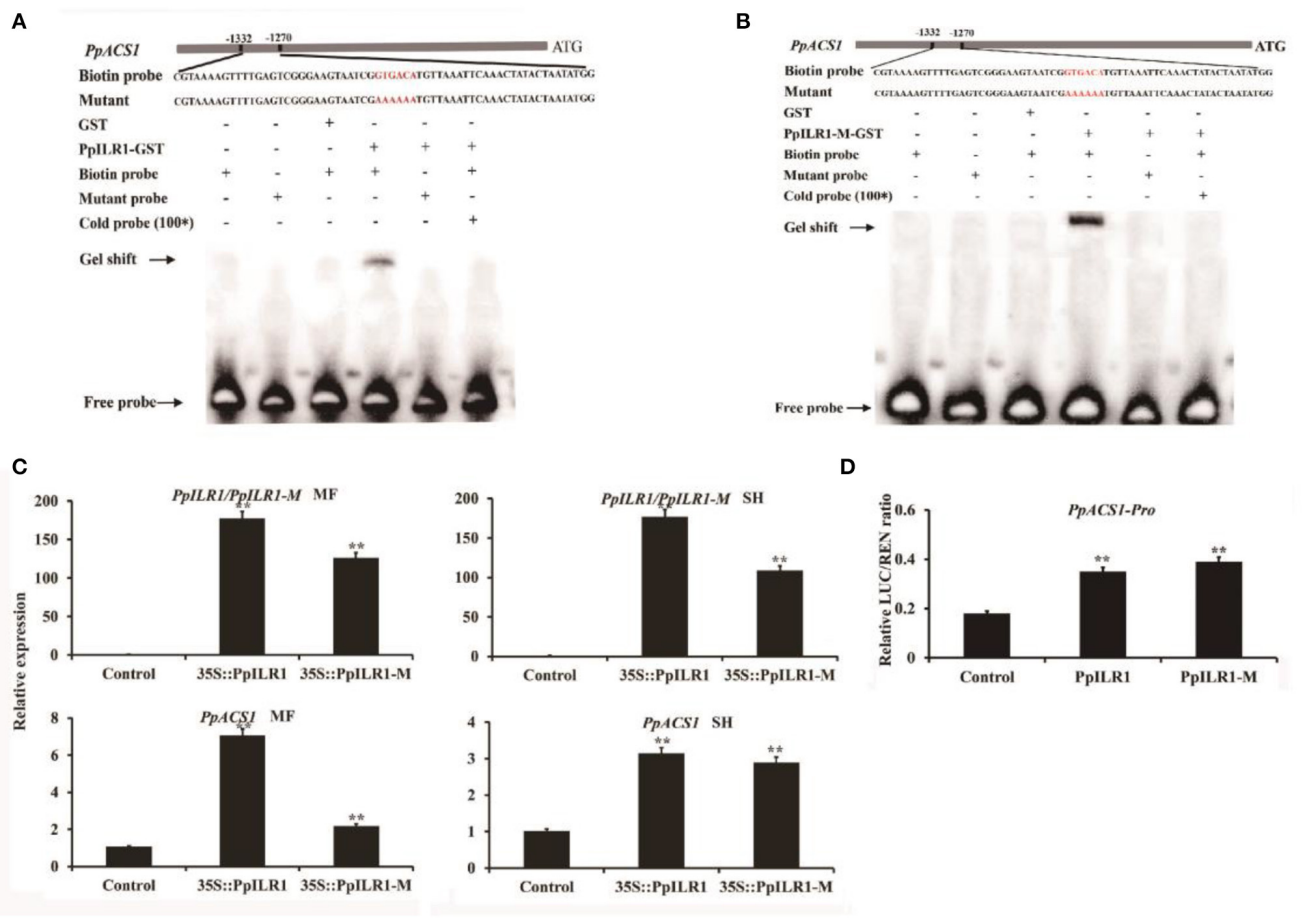
PpILR1 and PpILR1-M activate transcription of *PpACS1*. **(A)** Electrophoretic mobility shift assay (EMSA) showing that PpILR1 binds to the GTGACA motif in the *PpACS1* promoter fragments, but not to the poly-A mutant fragment. **(B)** EMSA showing that PpILR1-M binds to the GTGACA motif in the *PpACS1* promoter. **(C)** Transient overexpression of *PpILR1* and *PpILR1-M* in MF- and SH-type peach fruits enhances the transcript levels of *PpACS1*. Transcript levels of *PpILR1, PpILR1-M*, and *PpACS1* in peach fruit infiltrated with the empty vector (control), 35S::PpILR1, and the 35S::PpILR1-M vector. **(D)** Dual-luciferase reporter assay analysis showing that PpILR1 and PpILR1-M activate the promoter of *PpACS1*. *Agrobacterium tumefaciens* harboring *PpILR1*/*PpILR1-M* or *PpACS1* promoter-driven firefly luciferase (LUC) reporter plasmids were infiltrated into tobacco leaves. Significantly higher LUC:REN ratios were obtained with the *PpILR1*/*PpILR1-M* vector than with the empty vector, indicating that PpILR1/PpILR1-M increases *PpACS1* promoter activity. Asterisks indicate significant differences (**P < 0.01; Student's *t*-test).

For the mutated aa (C to S) was located at 137, the binding between PpILR1_1−120*aa*_ and *PpACS1-P1* should not be affected. To confirm the interaction of PpILR1 and PpILR1-M with *PpACS1-P1*, the PpILR1 and PpILR1-M proteins were purified and used for EMSAs. The results showed that both PpILR1 and PpILR1-M bound to the GTGACA element, regardless of their hydrolase activity. When the GTGACA element was changed into AAAAAA, no binding was observed (Figures 4A,B). To confirm the transcriptional activation function of PpILR1 and PpILR1-M, transient expression assays were conducted in peach fruit harvested at the S3 stage. In agroinfiltration assays conducted using the SH-type peach fruit, both PpILR1 and PpILR1-M significantly increased the expression of *PpACS1* by ~3-fold compared with the empty vector control. In MF-type peach fruit, PpILR1-M increased the expression of *PpACS1* by 2-fold, whereas PpILR1 upregulated the expression of *PpACS1* by ~7-fold compared with the empty vector control (Figure 4C). To further investigate the transcriptional activation activity of PpILR1 and PpILR1-M *in planta*, transient promoter activation assays were carried out. The results showed that both PpILR1 and PpILR1-M increased *PpACS1* promoter activity by ~2-fold, as shown by the increase in LUC:REN ratio, compared with the empty vector control (Figure 4D). This suggests that both PpILR1 and PpILR1-M function as transcriptional activators, regardless of their hydrolase activity. To confirm that PpILR1 functions as a transcriptional activator by binding to the auxin-responsive element, six repeats of the GTGACA motif were cloned into the pGreen0800-LUC vector; the pGreen0800-LUC vector containing the AAAAAA element was used as a control. The results of the DLR assay showed that the LUC:REN ratio increased by ~4-fold compared with the control (Supplementary Figure 9).

### Effect of *PpILR1* Overexpression on Shoot Architecture

**Figure 5:**
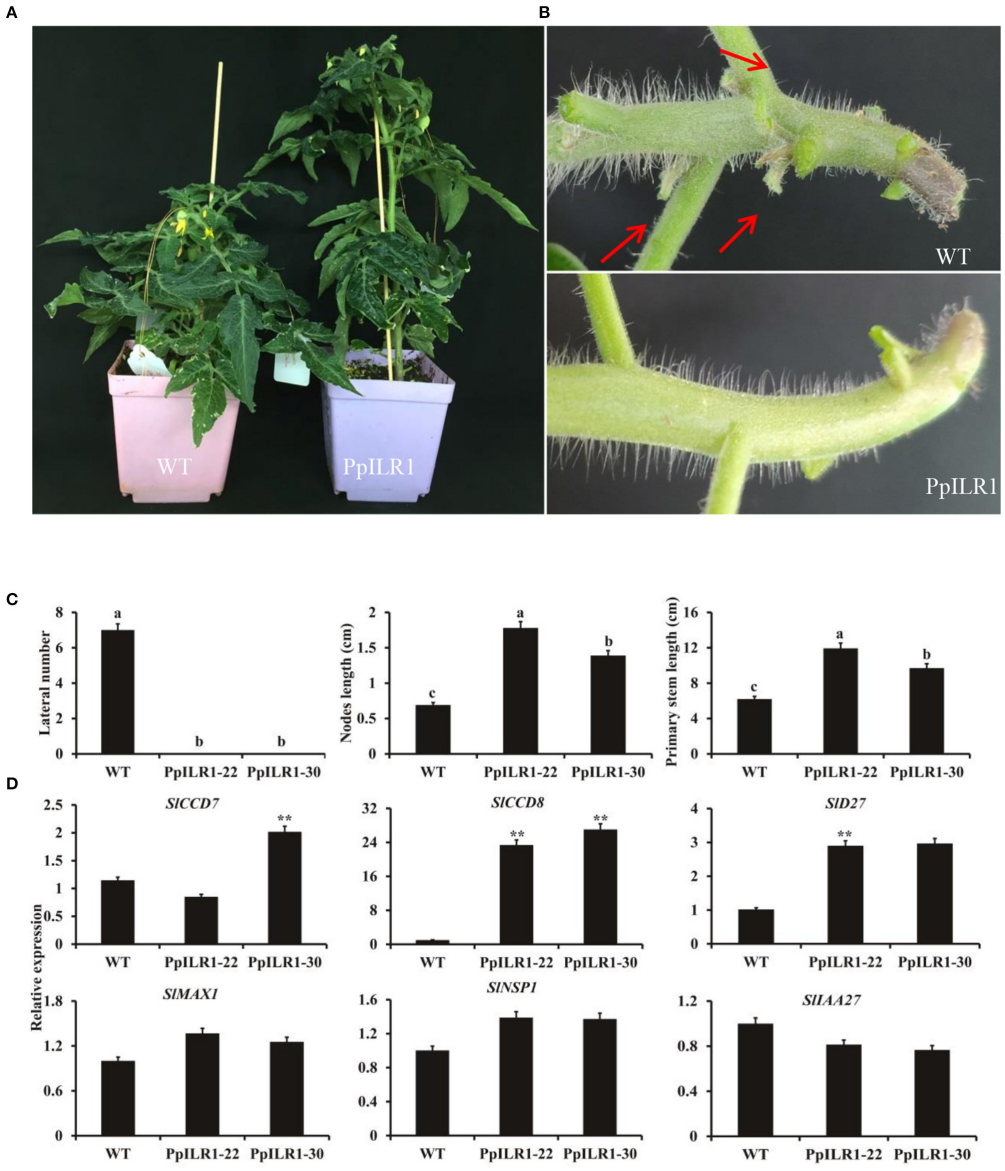
Altered vegetative growth phenotypes of *35S:PpILR1* overexpression tomato lines. **(A,B)** Photograph **(A)** and lateral branch number **(B)** of 7-week-old WT and *PpILR1*-overexpressing plants. **(C)** Close-up of 7-week-old WT and transgenic lines showing the lateral branch number, primary stem length, and internode length. **(D)** Expression of strigolactone biosynthesis genes in the axillary buds of WT, PpILR1-22, and PpILR1-30 plants. Asterisks indicate significant differences relative to the WT (*P* < 0.01; Student's *t*-test). Different letters indicate significant differences at *P* < 0.05.

To study the biological function of PpILR1 in further detail, 14 independent transgenic tomato lines overexpressing *PpILR1* were generated (Supplementary Figure 10). *PpILR1* transcripts were detected in all 14 transgenic plants at different levels, but were not detected in the WT control (Supplementary Figure 10). Two transgenic tomato lines (PpILR1-22 and PpILR1-30) with relatively higher levels of *PpILR1* transcripts were selected for further analysis (Supplementary Figure 10). Compared with the WT, plants overexpressing *PpILR1* were taller (Figure 5A) and did not produce lateral shoots (Figures 5B,C). Furthermore, these transgenic lines displayed longer primary stem and internodes than the WT (Figure 5C). A previous study showed that strigolactones can regulate plant architecture and shoot branching in tomato (Vogel et al., 2009). Therefore, we analyzed expression of strigolactone biosynthesis genes, including *SlCCD7, SlCCD8, SlD27, SlMAX1, SlNSP1*, and *SlIAA27*, by qRT-PCR. The results showed that the expression levels of *SlCCD8* and *SlD27* were ~10- and 3-fold higher in transgenic lines than in the WT (Figure 5D).

### *PpILR1* Overexpression Lines Displayed a Suite of Ethylene Hypersensitive Phenotypes

Several potentially ethylene-regulated developmental processes were altered in the transgenic line PpILR1-22. For example, most of the flowers of line PpILR1-22 showed premature senescence and abscission of petals before full opening (Supplementary Figure 11A). Additionally, plants of line PpILR1-22 showed leaf and petiole epinasty (Supplementary Figure 11B). To determine whether these phenotypes were related to ethylene, we analyzed the expression level of ethylene biosynthesis genes in the floral abscission zone. Consistent with the increased ethylene production, the expression levels of ethylene biosynthesis genes *SlACS1A, SlACS2, SlACS4, SlACS6*, and *SlACO1* were 6-, 4-, 7-, 2.5-, and 3-fold higher, respectively, in line PpILR1-22 than in the WT (Supplementary Figure 11C).

### Effect of *PpILR1* Overexpression on Fruit Ripening

**Figure 6:**
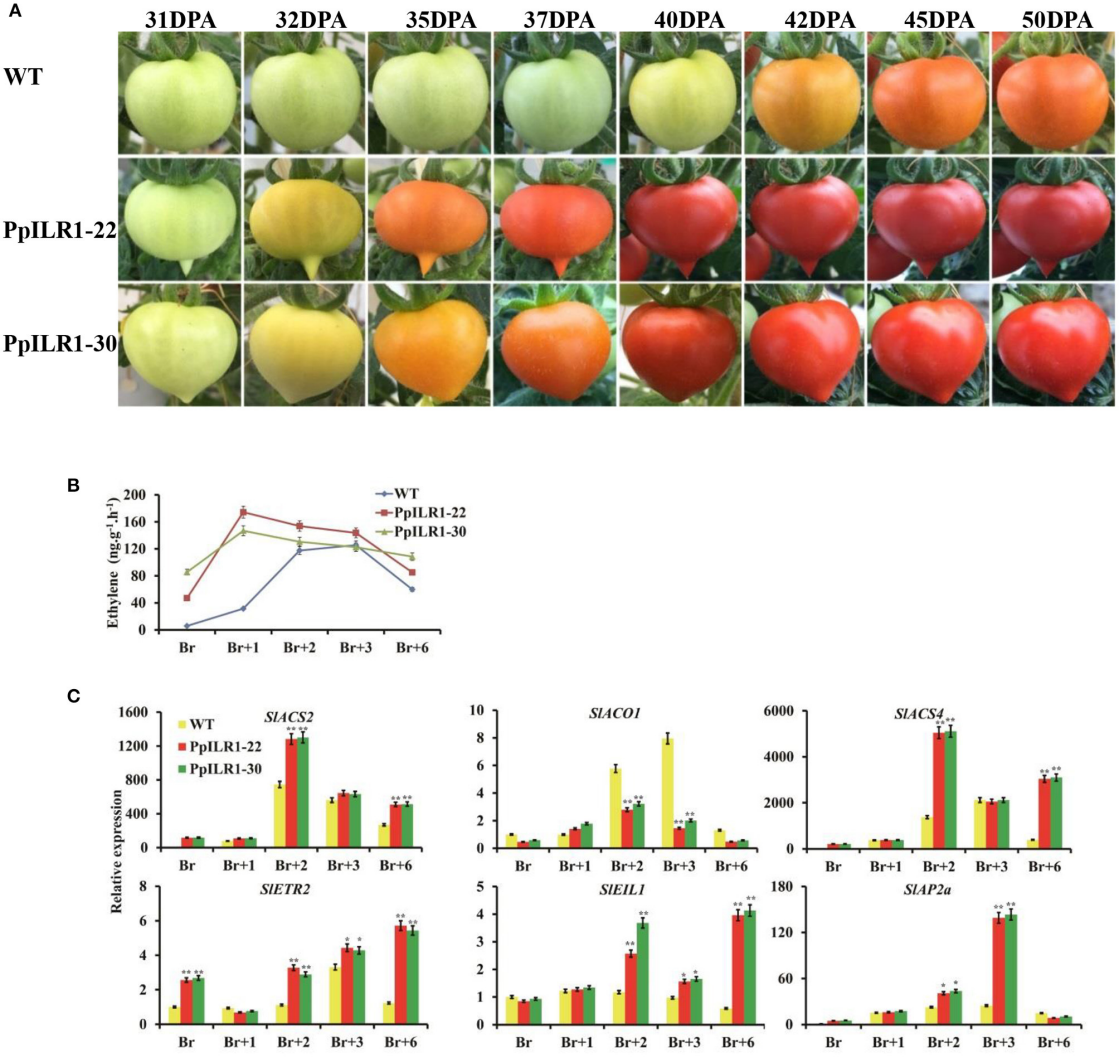
*PpILR1* overexpression in tomato accelerates fruit ripening. **(A)** Accelerated fruit ripening in two tomato transgenic lines, PpILR1-22 and PpILR1-30, compared with the WT. **(B)** Ethylene production in PpILR1-22, PpILR1-30, and the WT control. **(C)** Expression analysis of ethylene biosynthesis- and fruit ripening-related genes in PpILR1-22, PpILR1-30, and WT control fruit by qRT-PCR. Values represent the mean ± standard deviation of three biological replicates. Asterisks indicate significant differences between the WT and transgenic lines (*P < 0.05, **P < 0.01; Student's *t*-test).

Fruits of PpILR1-22 and PpILR1-30 transgenic lines ripened earlier than those of WT plants (Figure 6A). Comparison of PpILR1-22, PpILR1-30, and WT fruits at 28, 31, 32, 35, 37, 40, 42, and 45 days post-anthesis (dpa) revealed that fruits of PpILR1-22 and PpILR1-30 transgenic lines changed color ~10 days earlier than those of WT plants (Figure 6A). Additionally, ethylene production in PpILR1-22 and PpILR1-30 fruits was higher than that in WT fruits; peak ethylene levels were slightly higher, and occurred earlier, in transgenic fruits than in WT fruits (Figure 6B). The expression level of ethylene biosynthesis genes *SlACS2* and *SlACS4* in both transgenic lines was higher than that in WT fruits at Br+2 and Br+6 stages. Compared with the WT, fruits of PpILR1-22 and PpILR1-30 transgenic lines also showed higher expression of several ethylene signal transduction genes (*SlEIL1, SlEIL3, SlEIL4*, and *SlETR2*) at Br+2 and Br+6. Expression of the tomato ripening-related genes (*SlNOR, SlRIN, SlNR, SlFUL1, SlFUL2, and SlAP2a*) at the Br+3 stage (*P* < 0.05), and the cell wall degradation-related gene (*SlPG*) at the Br+2 stage (Figure 6C and Supplementary Figure 12), were also significantly higher. Taken together, these results suggest that overexpression of *PpILR1* accelerates fruit ripening in tomato by advancing and increasing production of ethylene, and by increasing expression of fruit ripening-related genes.

### Effect of PpILR1-M on Shoot Architecture and Fruit Ripening

The pK2GW7-PpILR1-M vector was constructed and used to transform tomato cultivar “Micro-Tom.” Two independent transgenic lines, PpILR1-M-6 and PpILR1-M-15, with higher expression levels of *PpILR1-M* (data not shown) were selected. Compared with the WT, transgenic lines PpILR1-M-6 and PpILR1-M-15 showed earlier fruit ripening (Supplementary Figure 13A). Additionally, fruits of lines PpILR1-M-6 and PpILR1-M-15 changed color ~5 days earlier than those of WT plants (Supplementary Figure 13A). However, the transgenic lines did not show any auxin related phenotypes, such as the lack of branching or an increase in primary stem length (Supplementary Figures 13B,C).

## Discussion

For decades, IAA-amido acid hydrolase enzymes were believed to perform a single function in plants, i.e., to convert auxin-amino acid conjugates such as IAA-Ala and IAA-Leu into free active IAA (LeClere et al., 2002; Schuller and Ludwig-Müller, 2006). Here, we show that an IAA-amido acid hydrolase enzyme, PpILR1, directly regulates expression of *PpACS1*, a rate limiting ethylene biosynthesis during fruit ripening. PpILR1 may regulate the ethylene biosynthesis pathway in two ways: directly by inducing expression of *PpACS1* via its transcriptional induction activity, and indirectly by releasing free active IAA from auxin-amino acid conjugates, which induces expression of *PpACS1* via the auxin signal transduction pathway. Overall, this study shows that PpILR1 is involved in auxin-ethylene crosstalk during peach fruit ripening.

In *B. rapa*, one of the two conserved Cys residues in the ILL2 enzyme, Cys139, is part of the active site and coordinates the metal cofactor (Smolko et al., 2016). According to the proposed model of the active site, Cys139 is a crucial amino acid residue in the binding pocket that potentially participates in metal binding. To prove this assumption, we generated mutants carrying a Cys137 to Ser137 substitution, and investigated the role of the Cys residue in regulating enzyme activity. The results showed that PpILR1 hydrolyzed IAA-Ala and IAA-Leu into free IAA, whereas the mutant protein PpILR1-M did not. This indicates that Cys137 is crucial for the hydrolase activity of PpILR1, consistent with a previous report (Smolko et al., 2016).

In plants, only about 5% of the total auxin concentration is in the free (active) form, while the remainder is stored in inactive forms, mostly as amino acid and sugar conjugates (Ludwig-Muller, 2011). IAA-Ala and IAA-Leu are proposed to be reversible storage forms of IAA in Arabidopsis (Ludwig-Muller, 2011; Korasick et al., 2013). These conjugates are not detected in MF and SH peach fruits at all growth stages, indicating that these storage forms are not involved in auxin metabolism in the peach fruit (Tatsuki et al., 2018). In our study, an increase in expression of *PpILR1* was accompanied by auxin accumulation, suggesting that IAA accumulates quickly during ripening of melting flesh peach fruits, owing to expression of *PpYUC11*, although a large amount of free IAA is converted into IAA-amino acid conjugates. These conjugates are transformed quickly into free IAA, which is accompanied by an increase in expression of *PpILR1*. This may explain why IAA-Ala and IAA-Leu were not detected in a previous study (Tatsuki et al., 2018). However, the mechanism by which auxin regulates expression of *PpILR1* needs further investigation.

Additionally, functioning as a transcription factor, we demonstrated a novel mechanism underlying the regulation of ethylene biosynthesis by an auxin-amino acid enzyme, thus showing additional crosstalk between auxin and ethylene in tomato and apple (Breitel et al., 2016; Yue et al., 2020). Moreover, our Y1H results showed that 1–120 aa of the PpILR1 protein interact with *PpACS1-P1*, not the conserved M20 region, suggesting different functions of the two domains of PpILR1. Our data suggest that the 1–120-aa domain of PpILR1 binds to the TGTCAC cis-element in the *PpACS1* promoter; however, the conserved region of this domain needs further investigation, and the conserved cis-element also needs to be confirmed.

Although ILR1 plays a positive role in ethylene production, we found that *PpILR1* expression was suppressed by ethylene and induced by 1-MCP treatment, as shown previously (Tadiello et al., 2016). Application of 1-MCP on peach fruits had almost no effect on the expression of auxin biosynthesis genes that regulate fruit ripening. The expression of two genes (*PpGH3.3* and *PpILR1*) was responsive to 1-MCP treatment (Supplementary Figure 5); 1-MCP treatment of peach fruits stimulated an increase in free auxin levels, possibly via upregulation of *PpILR1* and downregulation of *PpGH3.3*. This may explains why 1-MCP is not used as an effective post-harvest tool in the peach industry.

In this study, transgenic transgene lines overexpressing *PpILR1* displayed increased plant height, decreased shoot branching and reduced node number compared with the WT. Previously, tomato plants expressing an *SlCCD7* antisense construct displayed decreased strigolactone biosynthesis and increased shoot branching (Vogel et al., 2009). Another study showed that the strigolactone biosynthesis gene, *SlCCD8*, regulates shoot architecture (Kohlen et al., 2012). Guillotin et al. (2017) demonstrated that a tomato Aux/IAA gene, *SlIAA27*, which is involved in the crosstalk link between auxin and strigolactone pathways, regulates SLs biosynthesis via the regulation of *NSP1* and NSP1 target genes (*SlD27* and *SlMAX1*), but not via the regulation of *SlCCD7* and *SlCCD*8. In this study, we showed a new crosstalk link between auxin and strigolactone pathways; *PpILR1* possibly regulates strigolactone biosynthesis via the regulation of *SlCCD*8 and *SlD27*, but not that of *SlIAA27* and *SlNSP1*, although the detailed regulatory mechanism needs further investigation.

Auxin and ethylene play important roles in the tomato flower abscission zone. Pretreatment with 1-MCP and application of auxin can prevent pedicel abscission after flower removal. A previous study shows that expression of three *ILR* family genes is upregulated in tomato within 2 h after removal of the flower (Meir et al., 2001). In the current study, transgenic tomato lines overexpressing *PpILR1* levels displayed premature senescence and abscission before full blooming; however, if PpILR1 only converts auxin-amino acid conjugates into free active IAA, then maybe high concentrations of auxin would inhibit abscission before full blooming. Therefore, we propose that *PpILR1* acts as a transcriptional activator by directly regulating ethylene biosynthesis by controlling the expression of *SlACS2, SlACS4*, and other ethylene signal pathway genes. Moreover, transgenic tomato plants showed a suite of other ethylene hypersensitive phenotypes, including leaf and petiole epinasty and fruit ripening, in addition to exhibiting upregulated expression of genes involved in ethylene production and fruit ripening. This suggests that PpILR1 directly affects the expression of these genes, leading to ethylene related phenotypes. However, silencing *SlILL1, SlILL5*, and *SlILL6* genes individually resulted in significantly accelerated abscission in pedicel explants (Fu et al., 2019). This suggests that ILRs perform different functions in different species, and the function of ILR proteins needs to be characterized further in detail.

Recent studies indicate that certain proteins can function as transcriptional activators, in addition to performing enzyme activity in plants. For example, 5-enolpyruvylshikimate-3-phosphate synthase functions as a transcriptional repressor of genes in the phenylpropanoid pathway, in addition to performing its canonical biosynthetic function in the shikimate pathway (Xie et al., 2018). In rice (*Oryza sativa*), OsALDH2B1 primarily functions as a mitochondrial aldehyde dehydrogenase regulating male fertility, but it also acts as both a transcriptional repressor and activator, regulating a diverse range of biological processes involving brassinolide, G protein, jasmonic acid, and salicylic acid signaling pathways (Ke et al., 2020). Thus, it is possible that a greater number of genes, which were previously considered to encode an enzyme with one function, perform additional roles such as transcriptional activation and are, therefore, involved in multiple metabolic pathways.

## Data Availability Statement

The original contributions presented in the study are included in the article/Supplementary Material, further inquiries can be directed to the corresponding authors.

## Author Contributions

XW and ZW conceived the experiments. JM and YW collected plant materials and conducted the experiments. LD performed phenotyping. HL analyzed the data. J-LY, NN, and ZW wrote the manuscript. All authors contributed to the article and approved the submitted version.

## Conflict of Interest

The authors declare that the research was conducted in the absence of any commercial or financial relationships that could be construed as a potential conflict of interest.
