# Supplementary material for: Diverse Functions of IAA-Leucine Resistant PpILR1 Provide a Genic Basis for Auxin-Ethylene Crosstalk During Peach Fruit Ripening
Source: Front Plant Sci. 2021 May 12;12:655758. doi: 10.3389/fpls.2021.655758 (PMC8149794; doi:10.3389/fpls.2021.655758)
Supplement: Supplementary file 2 [file Table_2.DOCX]

| Assay | Genes Primer sequence | | | Restriction Site |
| --- | --- | --- | --- | --- |
|  | | Forward primer(5’-3’) | Reverse primer(5’-3’) |  |
| PCR | PpILR1 | ATGGGTTTCAATTTCACTTTCT | CTACTGAGTGTTAACATCATCATGG |  |
| RT-qPCR | PpILR1 | TCTTCCAATAGGAGCAGCACT | CATTTGTGCTTTAGTAAGACGG |  |
|  | Actin | GATTCCGGTGCCCAGAAGT | CCAGCAGCTTCCATTCCAA |  |
| Subcelluar  localization | PM999-PpILR1 | agcagatctatcgattctaga ATGGGTTTCAATTTCACTTTCT | tcctttgcccatggctctagaCTACTGAGTGTTAACATCATCATGG | BamHI  XbaI |
| YIH | PAbAi-PpACS1 | aaatgatgaattgaaaagcttGTTGATTGGAGGTGAAAGTGTGAG | atacagagcacatgcctcgagTTGCAAGCAAGCTATCAATTG | HindIII  XhoI |
|  | PABAi-PpACS1-P1 | aaatgatgaattgaaaagcttAATGTGCTTGAAACCAAACCAGC | atacagagcacatgcctcgagACATTTACTCCATTTCCAAAAGTTATG | HindIII  XhoI |
|  | PABAi-PpACS1-P2 | aaatgatgaattgaaaagcttGGCTTAATTAAAAGTCTTCCCAACTAT | atacagagcacatgcctcgagAAATTTAACGATATATCTATTTTCAAATCACA | HindIII  XhoI |
|  | PAbAi-PpACS1-P3 | aaatgatgaattgaaaagcttGTGTGTTTAAGGCGTTAAAAAATAAGG | atacagagcacatgcctcgagGAATTTTAAGTGAGGTTTGCAAGCA | HindIII  XhoI |
|  | AD-PpILR1 | gccatggaggccagtgaattcATGGGTTTCAATTTCACTTTCT | atgcccacccgggtggaattcCTACTGAGTGTTAACATCATCATGG | EcoRI |
| Y2H | AD- PpIAA1 | gccatggaggccagtgaattcATGAACATGCCACCGGATGC | atgcccacccgggtggaattcTTGTGGCAGAACCTGAGACGT | EcoRI |
|  | BD-PpILR1 | atggccatggaggccgaattcATGGGTTTCAATTTCACTTTCT | tcgacggatccccgggaattcCTACTGAGTGTTAACATCATCATGG | EcoRI |
| BiFC | PpERF2-GNE | GTGGATCCATGGCCTCAACCTCTTCCG | CCCTCGAGATAGCTCCAAAGGAGACTTTC | BamHI  XhoI |
|  | PpILR1-GCE | CTAGTGGATCCATGGGTTTCAATTTCACT | TACCCTCGAGCTGAGTGTTAACATCATCA | BamHI  XhoI |
| Dual LUC  assay | PpACS1 | ctatagggcgaattgggtaccGTTGATTGGAGGTGAAAGTGTGAG | tatatttttggcgtcttccatTTGCAAGCAAGCTATCAATTG | NcoI  KpnI |
| GST | PpILR1 | caggggcccctgggatccATGGGTTTCAATTTCACTTTCT | cggccgctcgagtcgaccCTACTGAGTGTTAACATCATCATGG | BamHI  SalI |

Table S2 Primers used in the manuscript.
