# Supplementary material for: Diverse Functions of IAA-Leucine Resistant PpILR1 Provide a Genic Basis for Auxin-Ethylene Crosstalk During Peach Fruit Ripening
Source: Front Plant Sci. 2021 May 12;12:655758. doi: 10.3389/fpls.2021.655758 (PMC8149794; doi:10.3389/fpls.2021.655758)
Supplement: Supplementary file 3 [file Table_3.DOCX]

Table S3 Probes in promoters of *PpACS1*. Auxin response element is indicated in red box, and probe is marked by biotin at 5’ terminus.

| Genes | Probes |
| --- | --- |
| *PpACS1* | CGTAAAAGTTTTGAGTCGGGAAGTAATCGGTGACATGTTAAATTCAAACTATACTAATATGG |
