# Supplementary material for: Diverse Functions of IAA-Leucine Resistant PpILR1 Provide a Genic Basis for Auxin-Ethylene Crosstalk During Peach Fruit Ripening
Source: Front Plant Sci. 2021 May 12;12:655758. doi: 10.3389/fpls.2021.655758 (PMC8149794; doi:10.3389/fpls.2021.655758)
Supplement: Supplementary file 4 [file Table_4.DOCX]

Table S4 Primers of tomato related genes used in the manuscript.

| Gene name | Gene ID | Primer Sequence |
| --- | --- | --- |
| SlNOR | Solyc10g006880 | TCCATGGGAACTCCCTGCTA |
|  |  | GATGTTGCAGCCCGATTTGG |
| SlTAGL1 | Solyc07g055920 | AGCTCATGCAAAAGAGGGAG |
|  |  | CGTAATTTGGCTGATGATGGTA |
| SlFUL1 | Solyc06g069430 | GGAAGAGTCCAGTTGAAGCGA |
|  |  | CCTCTCCATGCAGGAATCGT |
| SlCNR | Solyc02g077920 | ACATCCTTCTTGCCAGGTCG |
|  |  | GCCTTCGGCAACTCCTCTTA |
| SlRIN | Solyc05g012020 | CAGCTTGAACGTCAATTGGAT |
|  |  | CTTTGCTCACCACAATGCCA |
| SlAP2a | Solyc03g044300 | AACGGACCACAATCTTGAC |
|  |  | CTGCTCGGAGTCTGAACC |
| SlTAG1 | Solyc02g071730 | agctcttgctggaatgaaac |
|  |  | ggtcttgtctagggtaatgg |
| SlFUL2 | Solyc03g114830 | CGATGCTGAAGTTGGACTCA |
|  |  | TCCTTTCCATGCAAGAGTCA |
| Sl-EIL1 | Solyc06g073720 | CCTCAACAATATGTCCAGCCA |
|  |  | TCATCCTTTGCCCATCTTCAG |
| Sl-EIL3 | Solyc01g096810 | ACAGGACTTCAAGAAACAACCA |
|  |  | GTGTTGTGCTCATAGTTGATCTG |
| Sl-EIL4 | Solyc06g073730 | TATACCCTGATCGTTGTCCAC |
|  |  | TTACACTCATCTTGAGCACCA |
| Sl-ETR2 | Solyc07g056580 | TTGGAGGAATCAATGAGGGC |
|  |  | TCATTACGCGCACGAACAG |
| SlNR | Solyc09g075440 | TGCTGTTCGTGTACCGCTTT |
|  |  | TCATCGGGAGAACCAGAACC |
| SlETR5 | Solyc09g089610 | GTGCTCTGGGCCCTTCACTA |
|  |  | GAACTTACGCACCCTCAATGC |
| SlACS1A | Solyc08g081540 | GGTGCTCATGAAATGCTTG |
|  |  | TGGGACCAAAAAGGCATCA |
| SlACS2 | Solyc01g095080 | TGTTAGCGTATGTATTGACAACTGG |
|  |  | TCATAACATAACTTCACTTTTGCATTC |
| SlACS4 | Solyc05g050010 | CTCCTCAAATGGGGAGTACG |
|  |  | TTTTGTTTGCTCGCACTACG |
| SlACS6 | Solyc08g008110 | GCCACAAATGATGGACATGG |
|  |  | GAGGCTTTTGGGTTGTTGACT |
| SlACO1 | Solyc07g049530 | GCCAAAGAGCCAAGATTTGA |
|  |  | TTTTTAATTGAATTGGGATCTAA |
| SlPG | Solyc10g080210 | TCAAGGGCACAAGTGCAACAAAGG |
|  |  | TGCACGTAGCCTCTGATGGTTT |
| SlMAX1 | Solyc08g062950 | CGCCCTTAGTTGCCAGAGAA  GCCAACCAAACCCATGTTCC |
| SlNSP1 | Solyc03g123400 | CGACCCCATATCAGAGTGGC  CCATGGGTCCGCGTAGAAAT |
| SlIAA27 | Solyc03g120500 | CCAAAAAGAGGGAATGGAGGTT  TGTTCTCCCTTCATCATCATTTTTC |
| SlCCD7 | Solyc01g090660 | AGCCAAGAATTCGAGATCCC  GGAGAAAGCCCACATACTGC |
| SlCCD8 | Solyc08g066650 | CCAATTGCCTGTAATAGTTCC  GCCTTCAACGACGAGTTCTC |
| SlD27 | Solyc09g065750 | TGTTCTTCTTCATGCAGGCAAAT  GCTGTTGCAATCTGCTTGGT |
