# Supplementary material for: Diverse Functions of IAA-Leucine Resistant PpILR1 Provide a Genic Basis for Auxin-Ethylene Crosstalk During Peach Fruit Ripening
Source: Front Plant Sci. 2021 May 12;12:655758. doi: 10.3389/fpls.2021.655758 (PMC8149794; doi:10.3389/fpls.2021.655758)
Supplement: Supplementary file 5 [file Table_5.DOCX]

Table S5 Y1H screening results.

| NO | Accession number | Gene annotation |
| --- | --- | --- |
| 1 | Prupe.4G009800 | NONE |
| 2 | Prupe.6G219700 | NADH-UBIQUINONE OXIDOREDUCTASE |
| 3 | Prupe.5G098100 | GST-CONTAINING FLYWCH ZINC-FINGER PROTEIN |
| 4 | Prupe.6G141100 | HEVEIN-LIKE PREPROPROTEIN |
| 5 | Prupe.4G050800 | Dormancy/auxin associated protein |
| 6 | Prupe.3G011600 | NONE |
| 7 | Prupe.2G301600 | HEAVY-METAL-ASSOCIATED DOMAIN-CONTAINING PROTEIN |
| 8 | Prupe.8G223400 | 12S SEED STORAGE PROTEIN CRA1-RELATED |
| 9 | Prupe.3G230300 | Wound-induced protein (DUF3774) |
| 10 | Prupe.5G076300 | HEMOGLOBINASE FAMILY MEMBER |
| 11 | Prupe.8G202900 | CYTOCHROME B5 ISOFORM B |
| 12 | Prupe.2G207100 | CYSTEINE PROTEASE FAMILY C1-RELATED |
| 13 | Prupe.4G271700 | TPR repeat (TPR_11) |
| 14 | Prupe.1G202200 | 60S RIBOSOMAL PROTEIN L8 |
| 15 | Prupe.3G228300 | EQUILIBRATIVE NUCLEOTIDE TRANSPORTER 2 |
| 16 | Prupe.8G067300 | GLUTAREDOXIN-RELATED PROTEIN 5, MITOCHONDRIAL |
| 17 | Prupe.7G049200 | PROTEIN EARLY RESPONSIVE TO DEHYDRATION 15 |
| 18 | Prupe.8G034100 | ABA/WDS induced protein |
| 19 | Prupe.3G053300 | CHARGED MULTIVESICULAR BODY PROTEIN |
| 20 | Prupe.8G099800 | NONE |
| 21 | Prupe.2G281300 | RIBOSOMAL PROTEIN S15P/S13E |
| 22 | Prupe.8G034100 | ABA/WDS induced protein |
| 23 | Prupe.7G044900 | Phosphopantothenoylcysteine decarboxylase / N-((R)-4'-phosphopantothenoyl)-L-cysteine carboxy-lyase |
| 24 | Prupe.1G356400 | Dehydrin |
| 25 | Prupe.7G100000 | IAA-AMINO ACID HYDROLASE ILR1 |
| 26 | Prupe.7G214600 | 60S RIBOSOMAL PROTEIN L28 |
| 27 | Prupe.8G140800 | Zinc-binding |
| 28 | Prupe.2G294100 | CYTOCHROME B561-RELATED |
| 29 | Prupe.2G193100 | CALCIUM-BINDING PROTEIN KIC |
| 30 | Prupe.5G100500 | Tyrosine--tRNA ligase / Tyrosyl-tRNA synthetase |
| 31 | Prupe.3G286400 | ADENINE NUCLEOTIDE ALPHA HYDROLASES-LIKE SUPERFAMILY PROTEIN-RELATED |
| 32 | Prupe.6G060500 | JOSEPHIN 1, 2 |
