# Supplementary material for: Diverse Functions of IAA-Leucine Resistant PpILR1 Provide a Genic Basis for Auxin-Ethylene Crosstalk During Peach Fruit Ripening
Source: Front Plant Sci. 2021 May 12;12:655758. doi: 10.3389/fpls.2021.655758 (PMC8149794; doi:10.3389/fpls.2021.655758)
Supplement: Supplementary file 7 [file Table_7.DOCX]

Table S7 Hydrolysis experiment of PpILR1and PpILR-M protein.

| Protein (ng/mL) | Substrate (50mM) | Hydrolysis products | |
| --- | --- | --- | --- |
|  |  | PpILR1 | PpILR1-M |
|  |  | IAA(mg.Kg^-1^) | IAA(mg.Kg^-1^) |
| 2 | IAA-Ala | 0.222 | 0 |
| 10 |  | 2.618 | 0 |
| 20 |  | 5.4645 | 0 |
| 2 | IAA-Leu | 1.0415 | 0.4225 |
| 10 |  | 7.058 | 0.3875 |
| 20 |  | 11.5210 | 0.387 |
