# Supplementary material for: Diverse Functions of IAA-Leucine Resistant PpILR1 Provide a Genic Basis for Auxin-Ethylene Crosstalk During Peach Fruit Ripening
Source: Front Plant Sci. 2021 May 12;12:655758. doi: 10.3389/fpls.2021.655758 (PMC8149794; doi:10.3389/fpls.2021.655758)
Supplement: Supplementary file 9 [file Data_Sheet_2.PDF]

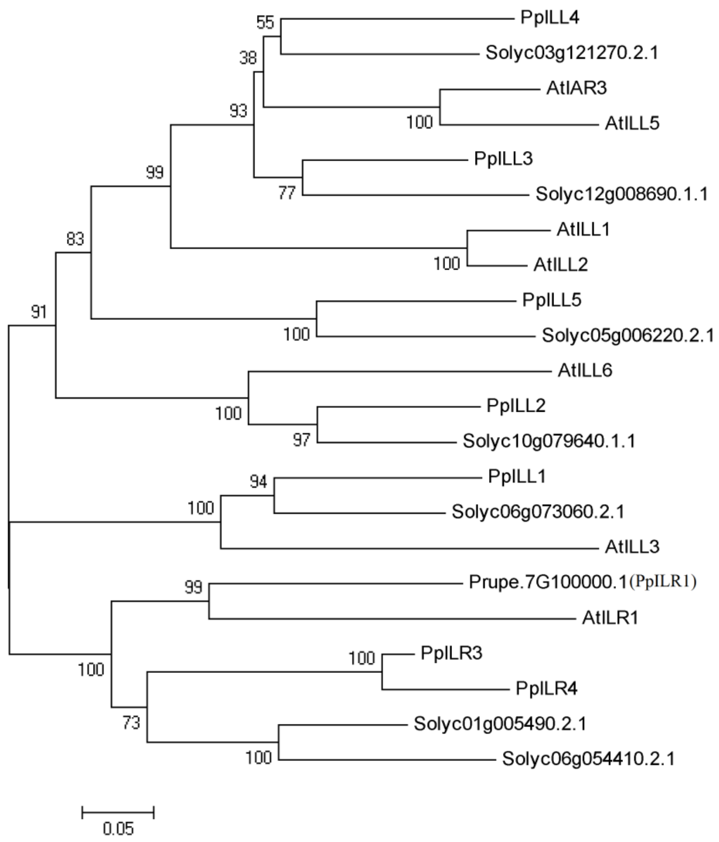

Fig. S2. Phylogenetic analysis of PpILR1, AtILR1，AT3G02875；AtIAR3，AT1G51760；AtILL1，AT5G56650；AtILL2，AT5G56660；AtILL3，AT5G54140；AtILL5，AT1G51780；AtILL6，AT1G44350.
