# Supplementary material for: Diverse Functions of IAA-Leucine Resistant PpILR1 Provide a Genic Basis for Auxin-Ethylene Crosstalk During Peach Fruit Ripening
Source: Front Plant Sci. 2021 May 12;12:655758. doi: 10.3389/fpls.2021.655758 (PMC8149794; doi:10.3389/fpls.2021.655758)
Supplement: Supplementary file 10 [file Data_Sheet_3.PDF]

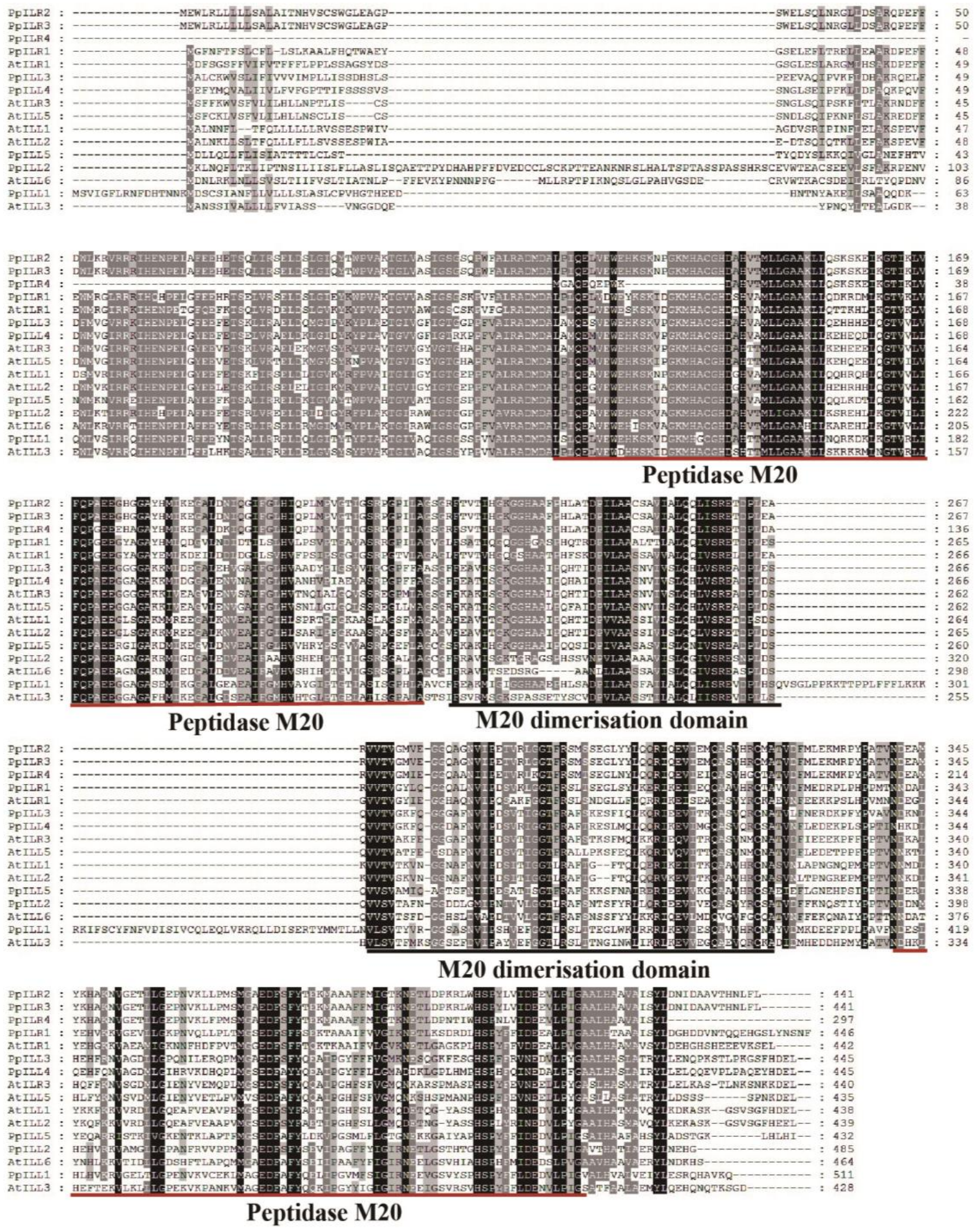

Fig. S3. Conservation of M20 domain structures analysis in peach ILL proteins. PpILR1: ppa005951m, PpILR2: ppa005836m, PpILR3: ppa005836m, PpILR4: ppa025680m, PpILL1: ppa006515m, PpILL2: ppa008809m, PpILL3: ppa005751m, PpILL4: ppa005752m, PpILL5: ppa006982m, AtILR1:AT3G02875, AtIAR3: AT1G51760, AtILL1: AT5G56650, AtILL2: AT5G56660, AtILL3: AT5G54140, AtILL5: AT1G51780, AtILL6:AT1G44350.
