# Supplementary material for: Diverse Functions of IAA-Leucine Resistant PpILR1 Provide a Genic Basis for Auxin-Ethylene Crosstalk During Peach Fruit Ripening
Source: Front Plant Sci. 2021 May 12;12:655758. doi: 10.3389/fpls.2021.655758 (PMC8149794; doi:10.3389/fpls.2021.655758)
Supplement: Supplementary file 11 [file Data_Sheet_4.PDF]

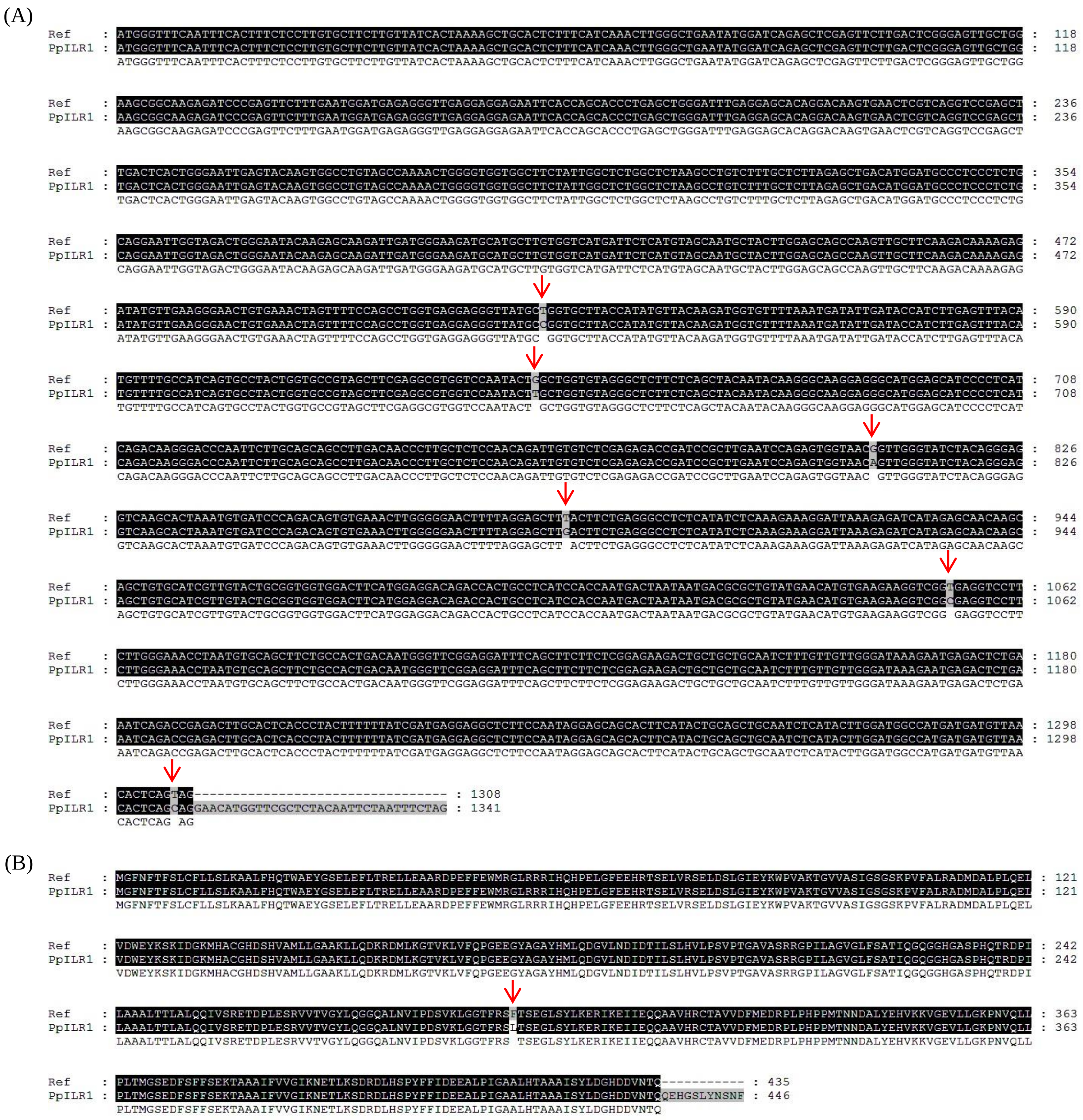

Fig. S4. Sequence alignment. (A) Base alignment of Ref and PpILR1. (B) Amino acid alignment of Ref and PpILR1. Ref refer to the reference genome sequence, PpILR1 refer to the sequence we cloned. The red arrows point to the mutation site.
