# Supplementary figures and images for: Diverse Functions of IAA-Leucine Resistant PpILR1 Provide a Genic Basis for Auxin-Ethylene Crosstalk During Peach Fruit Ripening
Source: Front Plant Sci. 2021 May 12;12:655758. doi: 10.3389/fpls.2021.655758 (PMC8149794; doi:10.3389/fpls.2021.655758)

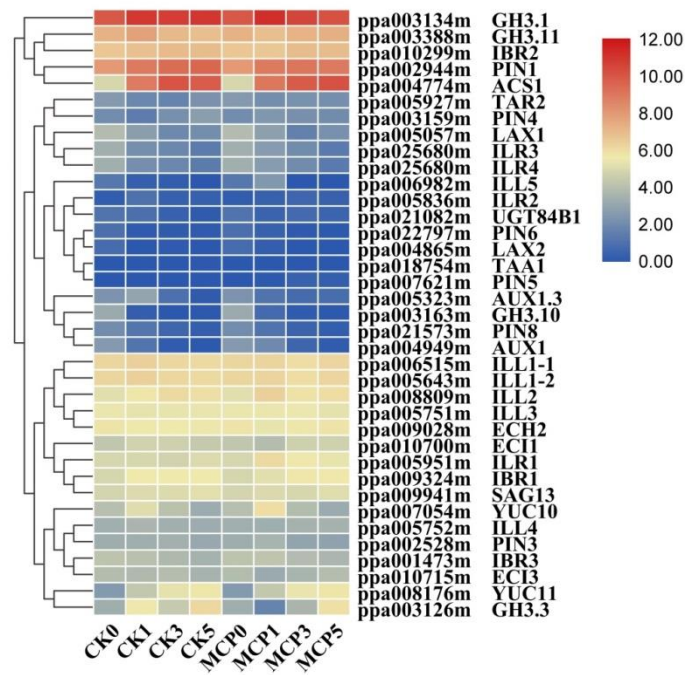

Fig. S5. Heatmap analysis of auxin-homeostasis-related genes in peach after treated with 1-MCP.

Supplement: Supplementary file 12 [file Data_Sheet_5.PDF]

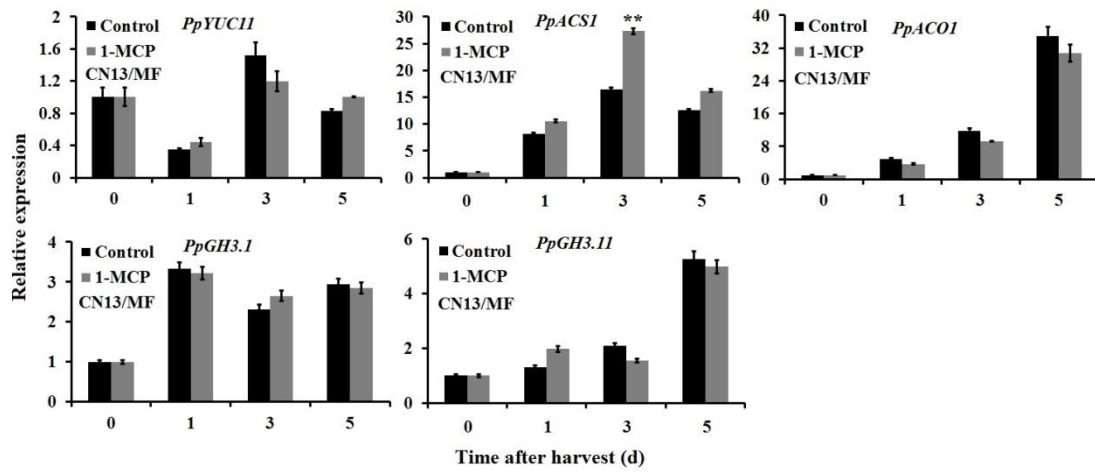

Fig. S6. The expression profile of *PpYUC11*, *PpACSI*, *PpACO1*, *PpGH3.1* and *PpGH3.11* in 'CN13' after treated with 1-MCP.

Supplement: Supplementary file 13 [file Data_Sheet_6.PDF]
