# Supplementary material for: Diverse Functions of IAA-Leucine Resistant PpILR1 Provide a Genic Basis for Auxin-Ethylene Crosstalk During Peach Fruit Ripening
Source: Front Plant Sci. 2021 May 12;12:655758. doi: 10.3389/fpls.2021.655758 (PMC8149794; doi:10.3389/fpls.2021.655758)
Supplement: Supplementary file 14 [file Data_Sheet_7.PDF]

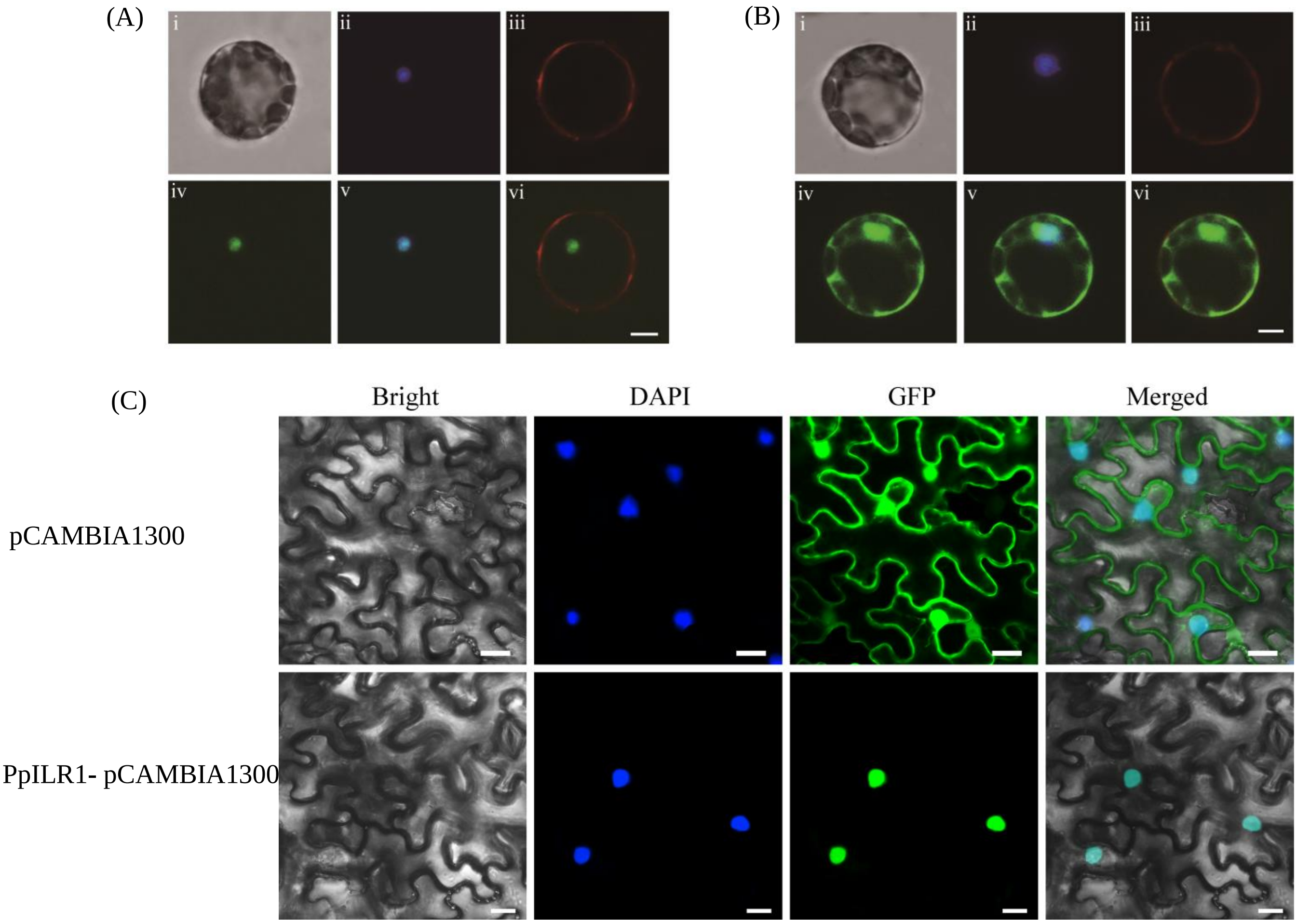

Fig. S7. Subcellular localization of PpILR1. (A) PM999-GFP (B) PpILR1-pM999. i visible light, ii DAPI, iii Dil, iv GFP, v GFP+DAPI merge, vi GFP+Dil merge. Scare bar, 5 um. (C) Subcellular localization of PpILR1 in tobacco leaves, PpILR1-Pcambia1300 were infiltrated into tobacco leaves via A. tumefaciens strain GV3101, and empty vector was used as negative control, Scare bar, 25 um.
