# Supplementary material for: Diverse Functions of IAA-Leucine Resistant PpILR1 Provide a Genic Basis for Auxin-Ethylene Crosstalk During Peach Fruit Ripening
Source: Front Plant Sci. 2021 May 12;12:655758. doi: 10.3389/fpls.2021.655758 (PMC8149794; doi:10.3389/fpls.2021.655758)
Supplement: Supplementary file 15 [file Data_Sheet_8.PDF]

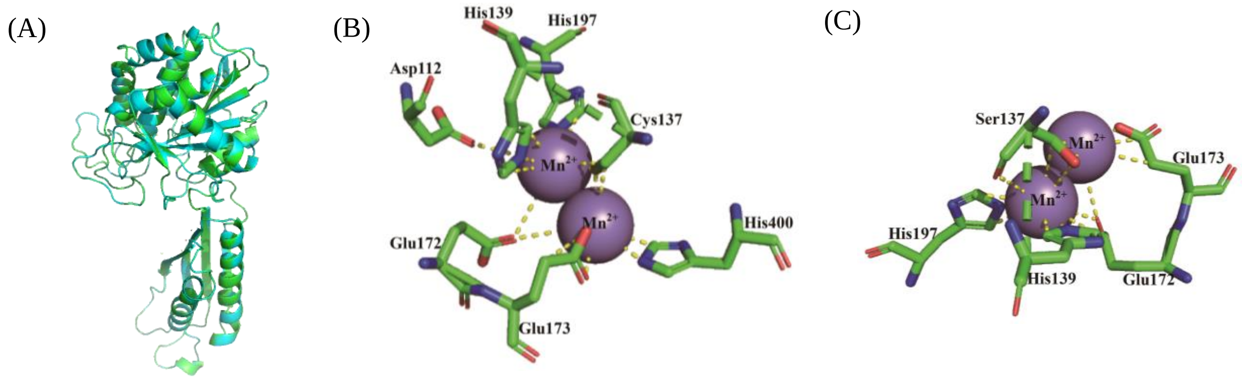

Fig. S8. Analysis of protein structure. (A) Alignment of PpILR1 (green) and PpILR1-M protein structure (blue). (B) Mn^{2+} surrounded by the PpILR1 protein. All residues within 4 Å ofmetal ions are displayed. The lower Mn^{2+} ion, which is closer to active site entrance, was surrounded by Glu172, Glu173, His400. The upper Mn^{2+} ion was surrounded by Asp112, Cys137, His139, His197. (C) Mn^{2+} surrounded by the PpILR1 protein. The Mn^{2+} ion was surrounded by Ser137, His139, Glu172, Glu173, His197.
