# Supplementary material for: Diverse Functions of IAA-Leucine Resistant PpILR1 Provide a Genic Basis for Auxin-Ethylene Crosstalk During Peach Fruit Ripening
Source: Front Plant Sci. 2021 May 12;12:655758. doi: 10.3389/fpls.2021.655758 (PMC8149794; doi:10.3389/fpls.2021.655758)
Supplement: Supplementary file 16 [file Data_Sheet_9.PDF]

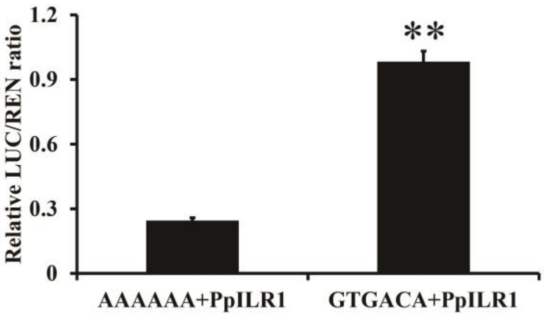

Fig. S9. The GTGACA (AAAAAA) element in the *PpACS1-P1* promoter was repeated six times. The oligonucleotides with partially complementary sequences were annealed, and then was used to cloning into the pGreenII0800-LUC vector which already containing the *PpACS1-P1* promoter.
