# Supplementary material for: Diverse Functions of IAA-Leucine Resistant PpILR1 Provide a Genic Basis for Auxin-Ethylene Crosstalk During Peach Fruit Ripening
Source: Front Plant Sci. 2021 May 12;12:655758. doi: 10.3389/fpls.2021.655758 (PMC8149794; doi:10.3389/fpls.2021.655758)
Supplement: Supplementary file 17 [file Data_Sheet_10.PDF]

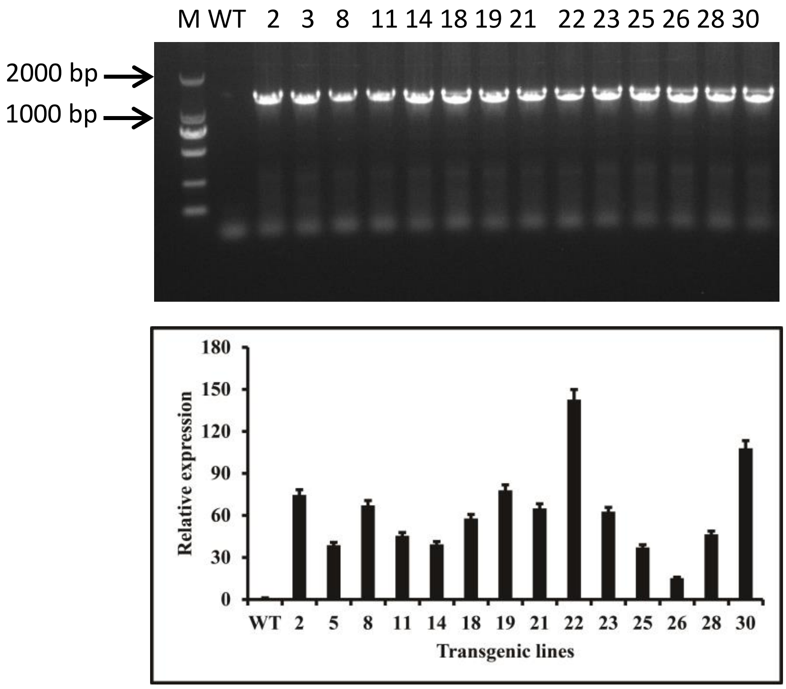

Figure. S10. Identification of transgenic lines. (A) The transgenic lines was screened by PCR. M: Marker 2000bp. (B) The level of PpILR1 transcripts in 14-day-old plants of WT and 35S:PpILR1 lines were assessed by qRT-PCR.
