# Supplementary material for: Diverse Functions of IAA-Leucine Resistant PpILR1 Provide a Genic Basis for Auxin-Ethylene Crosstalk During Peach Fruit Ripening
Source: Front Plant Sci. 2021 May 12;12:655758. doi: 10.3389/fpls.2021.655758 (PMC8149794; doi:10.3389/fpls.2021.655758)
Supplement: Supplementary file 18 [file Data_Sheet_11.PDF]

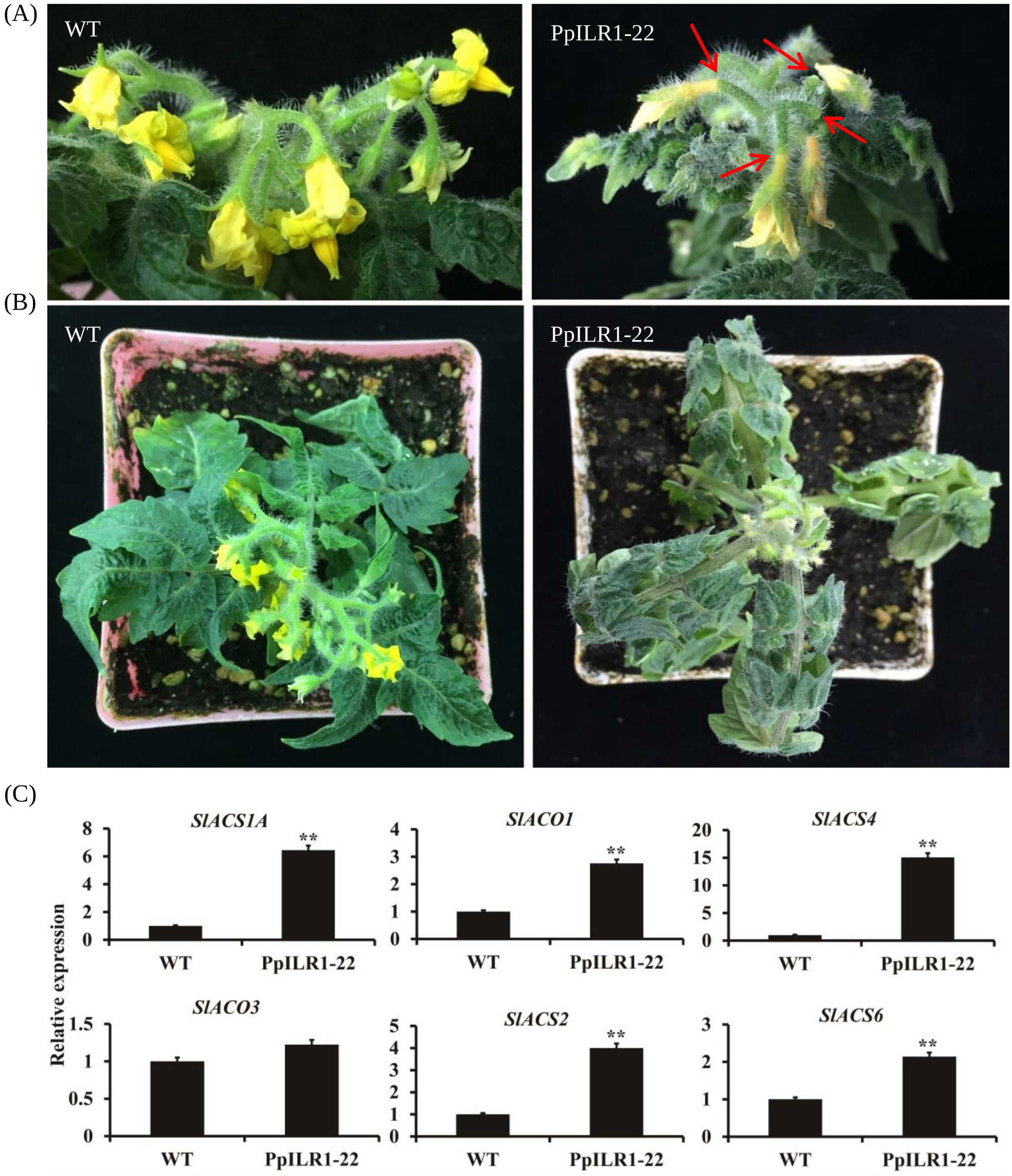

Figure. S11. Ethylene-related phenotype. (A) Comparing with WT, enhanced premature flower senescence was observed in PpILR1-22 line. (B) PpILR1-22 transgenic line showed petioles and leaves epinasty phenomenon compared with WT. (C) Ethylene biosynthesis genes was highly increased in PpILR-22 transgenic line. “**” represent significance at $p<0.01$, compared to WT based on t-test.
