# Supplementary material for: Diverse Functions of IAA-Leucine Resistant PpILR1 Provide a Genic Basis for Auxin-Ethylene Crosstalk During Peach Fruit Ripening
Source: Front Plant Sci. 2021 May 12;12:655758. doi: 10.3389/fpls.2021.655758 (PMC8149794; doi:10.3389/fpls.2021.655758)
Supplement: Supplementary file 19 [file Data_Sheet_12.PDF]

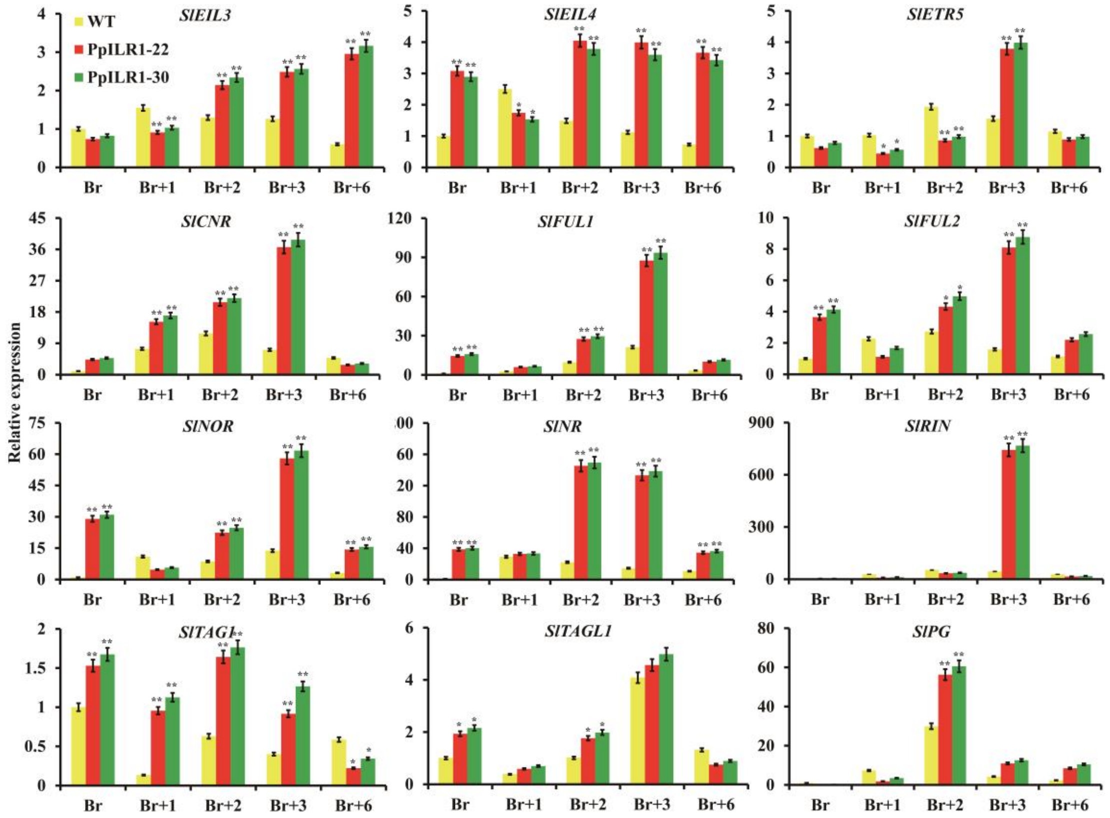

Fig. S12. PpILR1-overexpression sharply increased the expression level of ripening related genes. Values are means ± SD of three biological replicates, ** represent significance at p<0.01, compared to WT based on t-test.
