# Supplementary material for: Diverse Functions of IAA-Leucine Resistant PpILR1 Provide a Genic Basis for Auxin-Ethylene Crosstalk During Peach Fruit Ripening
Source: Front Plant Sci. 2021 May 12;12:655758. doi: 10.3389/fpls.2021.655758 (PMC8149794; doi:10.3389/fpls.2021.655758)
Supplement: Supplementary file 20 [file Data_Sheet_13.PDF]

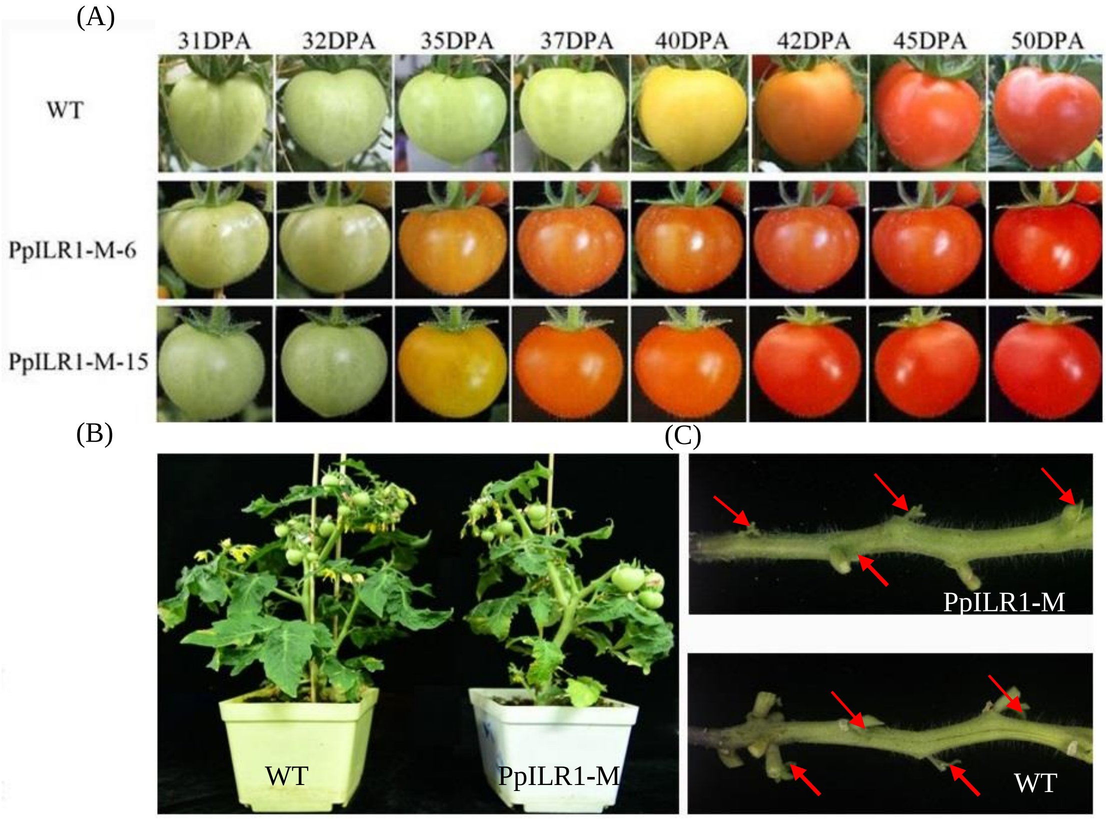

Fig. S13. Altered vegetative growth phenotypes in 35S:PpILR1-M overexpression tomato lines. (A)Two tomato transgenic lines, PpILR1-M-6 and PpILR1-M-15, showed an accelerated fruit ripening compared with WT. (B) Photograph of 7-week-old wild type and transgenic PpILR1-M plants. (C) Lateral branch number of WT, PpILR1-M of 7-week-old plants.
